# Chemotaxis in Densely Populated Tissue Determines Germinal Center Anatomy and Cell Motility: A New Paradigm for the Development of Complex Tissues

**DOI:** 10.1371/journal.pone.0027650

**Published:** 2011-12-01

**Authors:** Jared B. Hawkins, Mark T. Jones, Paul E. Plassmann, David A. Thorley-Lawson

**Affiliations:** 1 Department of Pathology, Tufts University School of Medicine, Boston, Massachusetts, United States of America; 2 The Bradley Department of Electrical and Computer Engineering, Virginia Tech, Blacksburg, Virginia, United States of America; INRA, France

## Abstract

Germinal centers (GCs) are complex dynamic structures that form within lymph nodes as an essential process in the humoral immune response. They represent a paradigm for studying the regulation of cell movement in the development of complex anatomical structures. We have developed a simulation of a modified cyclic re-entry model of GC dynamics which successfully employs chemotaxis to recapitulate the anatomy of the primary follicle and the development of a mature GC, including correctly structured mantle, dark and light zones. We then show that correct single cell movement dynamics (including persistent random walk and inter-zonal crossing) arise from this simulation as purely emergent properties. The major insight of our study is that chemotaxis can only achieve this when constrained by the known biological properties that cells are incompressible, exist in a densely packed environment, and must therefore compete for space. It is this interplay of chemotaxis and competition for limited space that generates all the complex and biologically accurate behaviors described here. Thus, from a single simple mechanism that is well documented in the biological literature, we can explain both higher level structure and single cell movement behaviors. To our knowledge this is the first GC model that is able to recapitulate both correctly detailed anatomy and single cell movement. This mechanism may have wide application for modeling other biological systems where cells undergo complex patterns of movement to produce defined anatomical structures with sharp tissue boundaries.

## Introduction

**Figure 1:**
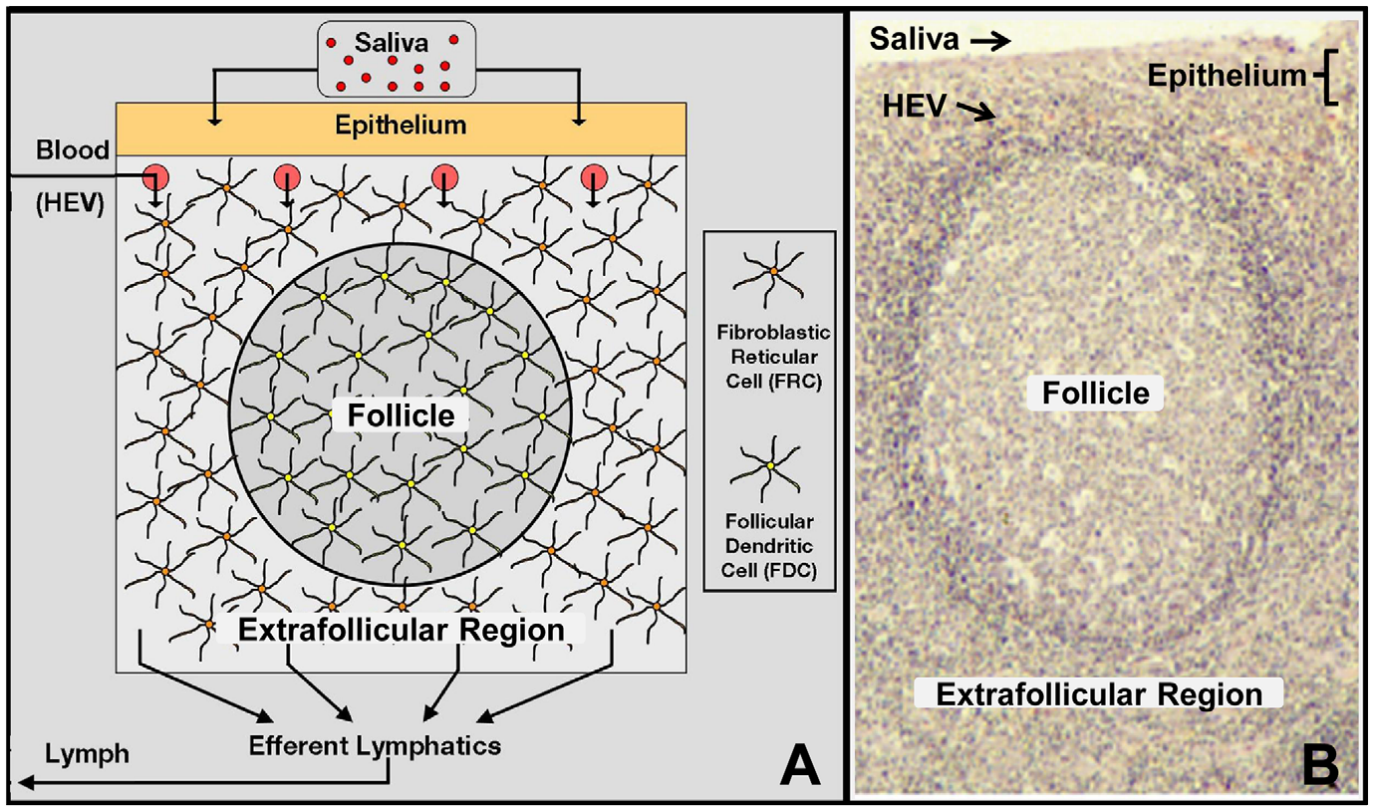
Basic Tonsil Unit. (**A**) The follicle and extrafollicular zones are distinguished by the presence of *FDCs* and *FRCs* respectively. *Lymphocytes* enter the mesh from the blood via the HEVs beneath the epithelium, move throughout the mesh and ultimately exit through efferent lymphatic vessels. (**B**) Histology of a human tonsil highlighting key architecture (image kindly supplied by Marta Perry). Video S1 displays the three-dimensional structure of an empty BTU rendered in PathSim2.

Germinal centers (GCs) are anatomically discrete, dynamic sites in the follicles of lymphoid tissue (Figure 1A) that are an essential component of the adaptive immune response (reviewed in [1], [2]). The development of GCs requires the carefully choreographed movement of multiple cell types within an environment that is densely packed with cells (Figure S1C). This movement is driven by gradients of chemokines. As such, GCs are a paradigm for understanding how cells migrate to form anatomically complex structures.

A primary follicle consists of naive B lymphocytes that enter the lymphoid tissue via extravasation from high endothelial venules (HEVs) and then migrate to the follicle (reviewed in [3]). Similarly, T-cells colonize the extrafollicular region. A T-cell dependent (TD) response is initiated through the interaction of antigen activated B-cells and T-cells [4], [5]. The result is the production of a small number of antigen specific GC founder B-cells. These cells proliferate rapidly within the follicle for ∼3 days (the initial expansion phase) [6], [7], displacing the naive B-cells which then form a characteristic structure around the GC termed the mantle zone (MZ) [6], [7], [8]. Although the MZ is discrete, the border with the GC is dynamic [9], [10]; there is no physical barrier preventing naive B-cells from entering the GC.

The end of the expansion phase marks the entrance into the next, competitive phase of the GC reaction (GCR) where cells display highly regulated migration as they undergo expansion, selection and death. At this point the GC resolves into two discrete zones, termed the light (LZ) and dark (DZ) zones, as the GC founder B-cells differentiate into centroblasts and centrocytes. Thus, mature GCs are highly ordered, with a characteristic structure consisting of a MZ surrounding the LZ and DZ.

In the cyclic re-entry model of GC development, a refinement of the classical model [11], centroblasts proliferate in the DZ where they undergo somatic hypermutation of their B-cell receptor genes [12], [13]. After each division they differentiate into centrocytes and migrate to the LZ [14], [15]. Here the centrocytes compete for access to antigen and T-cell help, both of which provide signals that are required for survival. Positively selected centrocytes in the LZ differentiate into centroblasts and return to the DZ, thereby completing one cell cycle. This process drives the selection of B-cells that produce high affinity antibodies [16]. Alternatively, positively selected cells in the LZ may differentiate further and leave the GC as output (plasma and memory B-cells).

At the single cell level, it has been observed that GC B-cells are extremely motile, undergoing a characteristic movement behavior termed “persistent random walk” (PRW), whereby the cells move directionally for a brief period of time before randomly changing direction [17]. The origins of this behavior are unknown and some authors have assumed that it is an intrinsic property of the cells [17]. Additionally, GC B-cells undergo a distinct rate of inter-zonal migration as they cycle between the LZ and DZ, and there is controversy regarding the interpretation of these rates. Hauser et al., in particular, have claimed that they are not consistent with the cyclic re-entry model of GC development [18].

Computer and mathematical modeling of dynamic systems are powerful investigative tools that have been applied in many scientific areas. Their successful application is dependent upon detailed and precise quantitative information. Such information about the movement of cells that constitute the GC has been provided by intra-vital multi-photon microscopy [9], [10], [18], making lymphocyte movement within lymph nodes a good candidate for analysis by modeling. Recently such an approach has been used in an attempt to explain the available data [17], [19]. However, none of these studies were able to explain and reconcile both the large scale anatomical features of the GC and the distinctive single cell movement behavior as emergent behaviors based on the known properties of the system.

In this study we have constructed and used a computer simulation (PathSim2) to model GC development. This model predicts that a single property of lymphoid tissue, namely directed chemotaxis, is sufficient to produce both the anatomical structure of the GC and, as emergent behavior, the characteristic movement of individual GC B-cells. This is only possible, however, when chemotaxis is modulated by the competition for space that arises as a consequence of the densely packed cellularity of the tissue.

## Results

### Accurate GC structure emerges from chemotaxis-driven competition for limited space

**Figure 2:**
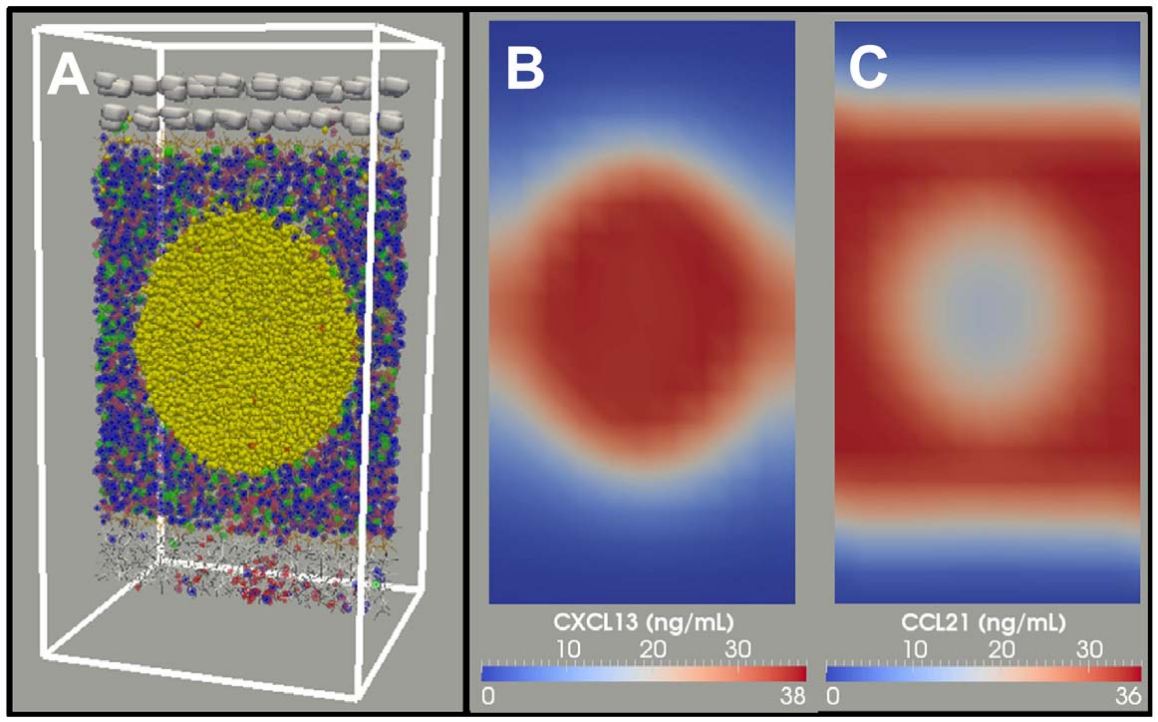
*Naive* homeostasis within the Basic Tonsil Unit. (**A**) At homeostasis, *naive T-cells* (blue, green) are distributed throughout the *FRC* populated extrafollicular zone while *naive B-cells* (yellow) are confined to the *FDC* populated follicle. For all PathSim2 graphical visualizations, we display a cross-sectional slice through the tissue, orientated with the epithelium facing up. Video S2 depicts an initially empty BTU developing into a primary follicle at homeostasis. (**B**) *FDC* produced *CXCL13* diffuses throughout the mesh but remains most concentrated within the follicle where it is produced. (**C**) *FRC* produced *CCL21* diffuses throughout the extrafollicular region and is less concentrated within the follicle.

PathSim2 (Pathogen Simulation 2) is an agent-based simulation that renders a small piece of tonsil lymphatic tissue, termed the Basic Tonsil Unit (BTU), consisting of a single follicle and all the relevant surrounding tissue necessary for its function (Figure 1, Video S1). For clarity, agent names and simulated chemokines will be written in *italics* to distinguish them from their real-life counterparts. We have based the default structure of our simulation on a variation of the cyclic re-entry model which takes into account the established observation that cells occasionally also divide in the LZ [10], [18], [20], [21] (see Methods for a detailed description of the simulation and Table S1 for a complete list of relevant agent parameters). As discussed below, this modification does not significantly impact the behavior of the simulation and is included primarily for biological accuracy. Figure 2A and Video S2 show the primary follicle at homeostasis. The localization of *naive lymphocytes* (*B* - yellow and *T* - blue) is driven solely by *chemokine* gradients that are compatible with biological data (Figure 2B-C; see Methods, Table S1).

**Figure 3:**
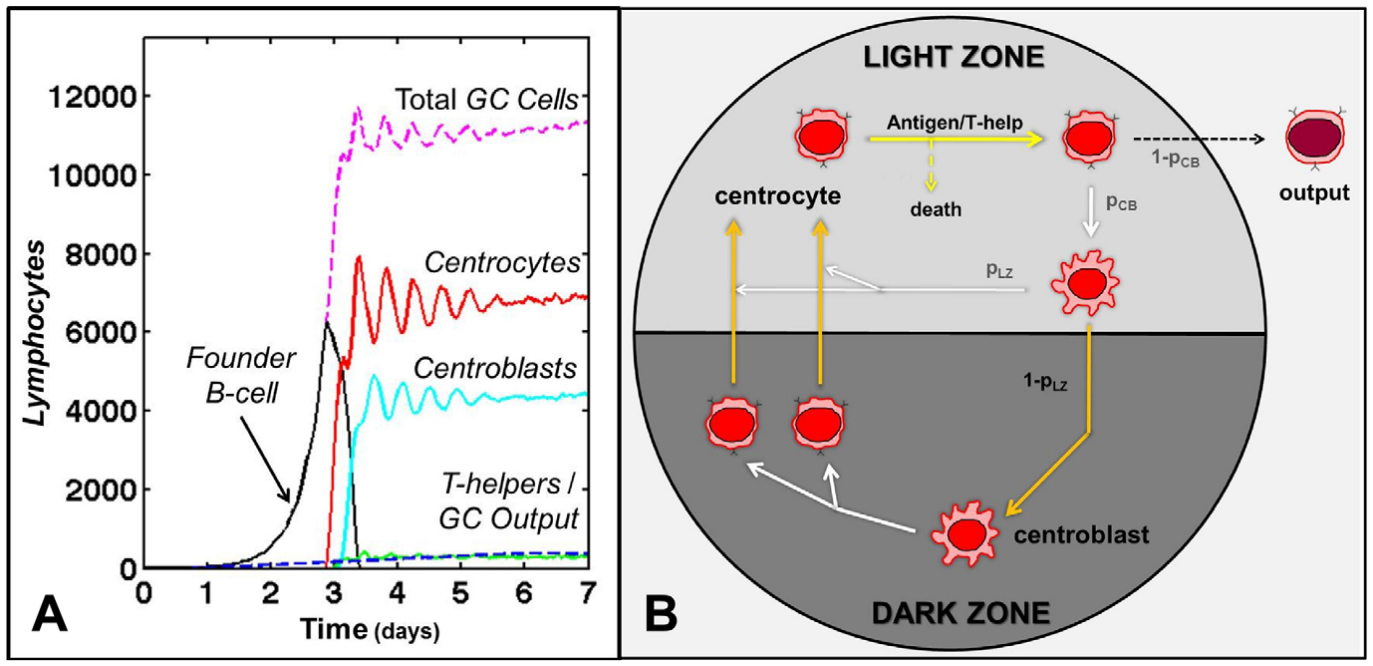
The modified cyclic re-entry model. This is the default state of our simulation. (**A**) The *GC-cell* populations over time. After seeding by 3 *GC founder B-cells*, a GC reaches peak size of ∼10^4^ cells in ∼3-4 days – the initial expansion phase. The mature GC populations reach a stable equilibrium after ∼6 days. *T-helpers* are shown in blue and *GC Output* is shown in green. The ratio of *centrocytes* to *centroblasts* at equilibrium (∼1.6∶1) is consistent with initial published observations that centrocytes outnumbered centroblasts at the peak of the GCR [24]. After reaching equilibrium the GC remains in this state. We do not model GC termination. All cell movement dynamics were measured in a GC at equilibrium. (**B**) *Centroblasts* divide in the DZ, each producing two *centrocytes* that cross from DZ → LZ. If successful in receiving positive selection, *centrocytes* primarily differentiate back into *centroblasts* (probability of p_CB_), and either remain in the LZ (probability of p_LZ_) or cross from LZ → DZ (probability of 1-p_LZ_) before initiating division, thus completing the cell cycle. A small percentage of selected *centrocytes* leaves the GC as output. Video S3 depicts the development of a GC starting from a primary follicle at homeostasis.

**Figure 4:**
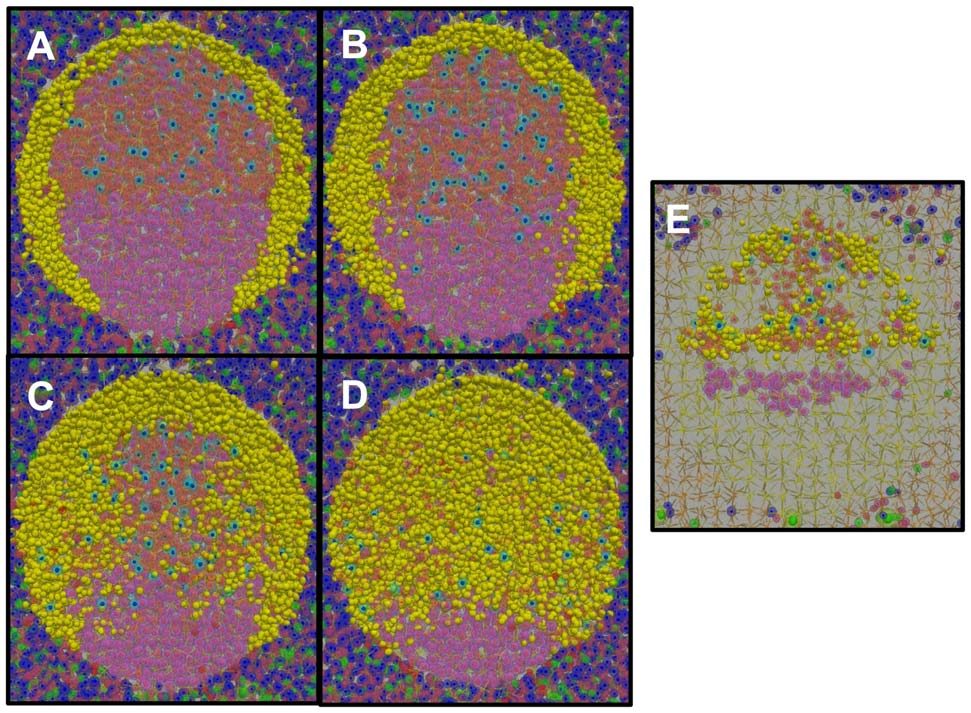
GC anatomy. (**A**) A cross-section through a mature GC at equilibrium. *Naive B-cells* (yellow) highlight the MZ surrounding the GC (orange/pink). *T-cells* (blue/green) are in the extrafollicular region, with the exception of *follicular T-helpers* in the LZ (light blue). (**A-D**) Mantle zone anatomy. In all panels, the *GC B-cell* chemotaxis parameter is 12 µm/min. Increasing the *naive B-cell* (shown in yellow) chemotaxis parameter [(A) 6 µm/min (B) 8 µm/min (C) 10 µm/min (D) 12 µm/min] directly affects the relative ability of *naive B-cells* to compete for space within the LZ, destroying the GC (shown in orange/pink) architecture. (**E**) GC anatomy depends on a dense environment. In a sparsely populated BTU (∼1/10 physiological level of lymphocytes), there is ample free space throughout the follicle and extra-follicular region. Under these conditions, *naive B-cells* (shown in yellow) are not driven from the secondary follicle by *GC B-cells* (*LZ cells* shown in orange, *DZ cells* shown in pink). This highlights the notion that there is no physical barrier restricting *naive B-cells* from entering a GC. In our system, it is the chemotaxis-driven competition for limited space that drives their exclusion, forming the MZ and maintaining the overall GC structure (LZ and DZ).

The GCR is initiated by seeding 3 *GC founder B-cells* into the follicle. As these *GC founder cells* proliferate and expand (Figure 3A, Video S3), they drive the *naive B-cells* away from the center of the *FDC* network of the developing secondary follicle, thereby forming the MZ (i.e., the yellow cells in Figure 4A). This occurs because *FDCs* alter their phenotype as the secondary follicle is formed [22], [23], such that *naive B-cells* are unable to move as efficiently as *GC B-cells*. Consequently, *naive B-cells* lose the chemotaxis-driven competition for limited space in the follicle, forcing them out of the GC. The dependence of MZ formation on both competition and the densely packed environment is demonstrated by two control experiments. In the first we varied the relative strength of *naive B-cell* chemotaxis. As this parameter approaches that of *GC B-cells*, *naive B-cells* begin to outcompete *GC B-cells* for space within the *FDC* network and MZ/GC integrity is lost (Figures 4B-D). Similarly, if the simulation is run in an environment that is sparsely populated with *lymphocytes* (∼1/10 the normal number), there is room for both *naive* and *GC B-cells* in the follicle, and a correctly positioned MZ does not form (Figure 4E).

**Figure 5:**
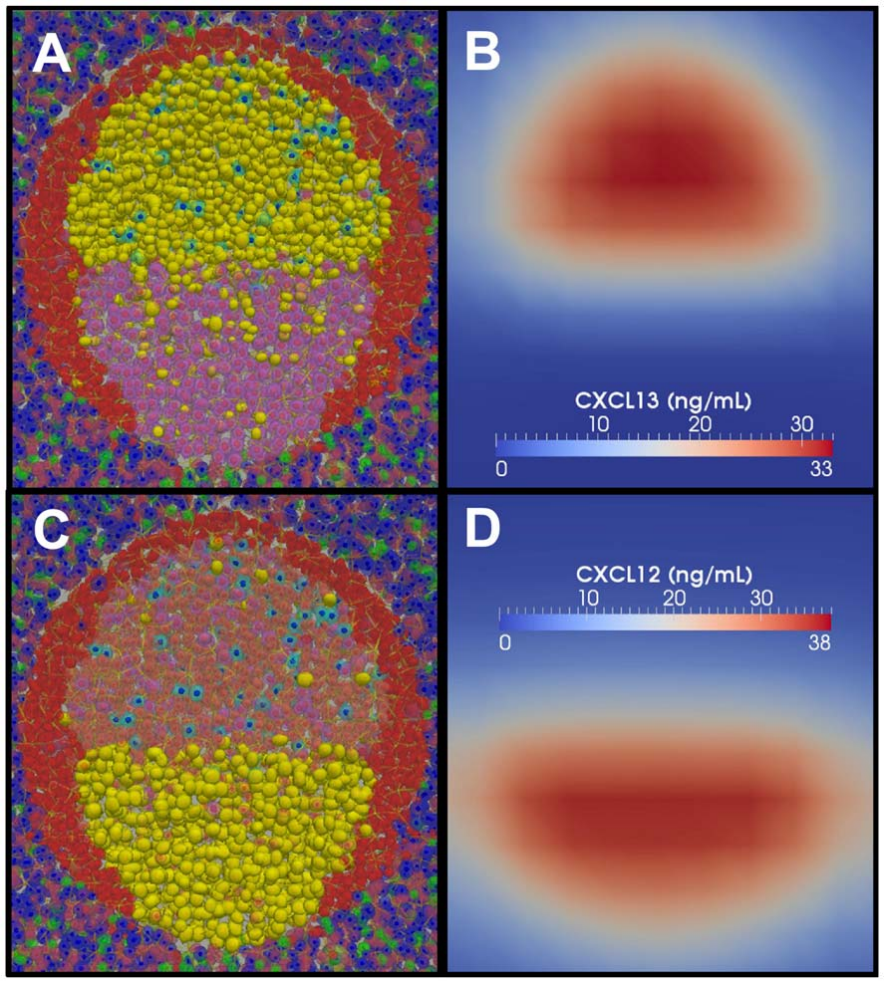
Light/Dark zone anatomy. A cross-section through a mature GC at equilibrium. (**A**) *Cells* in the LZ (*centrocytes*) are responsive to *CXCL13,* shown in (**B**). (**C**) *Cells* in the DZ (*centroblasts*) are primarily responsive to *CXCL12,* shown in (**D**). *B-cells* highlighted in yellow are following the corresponding *chemokine* gradients.

The distinctive feature of cyclic re-entry models is the movement of GC B-cells between functionally distinct DZs and LZs (Figure 3B,5), driven by regulated gradients of zonal *chemokines* (Figure 5B,D). We have recapitulated this behavior in our simulation, applying only *chemokine* gradients that are compatible with biological data (see Methods). Figure 5 shows a mature simulated GC at peak size and the *chemokine* gradients that produced it. Note that the ratio of *centrocytes* to *centroblasts* that we achieve (∼1.6∶1, Figure 3A) is consistent with the originally published observations that centrocytes outnumber centroblasts at the peak of the GCR [24] (see Methods).

The simulation accurately produces all of the characteristic features of a normal mature GC including appropriately sized and positioned MZ, LZ and DZ. Note that we also achieve the densely packed cellularity of the GC, correctly mimicking what is seen in vivo (Figure 1B,S1C; see Methods). We have performed a control simulation to test if a correctly structured GC is dependent upon the modulation of directed chemotaxis by this densely packed environment. In this control we removed the competition for space by allowing the same chemotaxis model to proceed under conditions of a sparsely populated BTU (∼1/10 *GC B-cells*). As may be seen in Figure 4E, the integrity of the MZ, LZ and DZ all become compromised. Our model predicts, therefore, that the chemotaxis-driven competition for space of cells in a densely packed environment is sufficient to drive the formation of an anatomically correct GC.

### Validation of simulation predictions

One way to validate simulations is to test if perturbations generate the same outcomes as observed experimentally. In our case, we used the simulation to predict the outcome of knocking out the gene for CXCR4, the receptor for CXCL12 (the DZ chemokine). Under these conditions in vivo, no discernible DZ is formed and LZ FDCs are present throughout the GC [25]. The result is that centroblasts become distributed throughout the GC.

**Figure 6:**
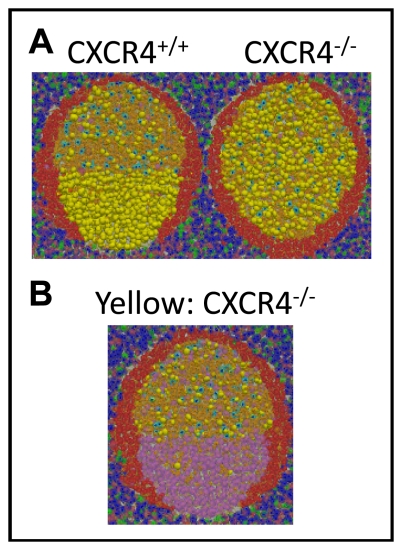
Validation of predicted GC anatomy. CXCR4, the receptor for CXCL12 (the DZ chemokine) is needed for entry into the DZ. (**A**) GC anatomy from CXCR4+/+ (WT) and CXCR4-/- (KO) GCs. The WT follicle contains both *LZ FDCs* and *DZ stromal cells*, while the KO follicle is comprised only of *LZ FDCs*. *Naive B-cells* are shown in red, *centrocytes* are shown in orange, and *centroblasts* are highlighted in yellow. This figure corresponds to an in vivo experiment (Figure 1B) originally published in [25]. (**B**) GC anatomy showing the location of CXCR4-/- *GC B-cells* (yellow) in a WT GC (∼1∶10 ratio of KO:WT *GC B-cells*). *Naive B-cells* are shown in red. This figure corresponds to an in vivo experiment (Figure 6B) originally published in [25].

In the simulation, we programmed a GC with *LZ FDCs* distributed throughout the follicle (Figure 6A). The consequence is the same as in vivo, namely *centroblasts* (labeled in yellow) are now dispersed throughout the GC. A small detail worthy of note from the in vivo KO GC is that the MZ is evenly distributed around the GC, whereas it tends to cap the LZ in the wild-type [25]. This behavior is also replicated in the simulated KO and is likely a consequence of altered chemokine gradients.

In the second study we simulated an experiment where mice were reconstituted such that they had CXCR4+/+ (CD45.2+) and CXCR4-/- (CD45.1+) *GC B-cells* at a ratio of ∼9/1. In an analogous in vivo experiment, a normal GC forms, but the CXCR4-/- cells are restricted to the LZ [25]. In the simulation (Figure 6B), CXCR4-/- *cells* are tagged in yellow. It is apparent that, just as in the in vivo experiment, a normal GC is formed, but the CXCR4-/- *cells* are restricted to the LZ.

The most important conclusions from these studies is that they demonstrate directly that the generation of structures like the LZ and DZ in our simulation is not a consequence of some pre-programmed behavior that is intrinsic to the *GC B-cells* and is telling them where to go. Rather, it is a consequence of differential responsiveness to *chemokines* and, just like in vivo, if that responsiveness is changed, then correct anatomy is disrupted.

### PRW is an emergent behavior of *GC B-cells* in the simulation

We have demonstrated that directed chemotaxis and competition for space are sufficient to produce accurate, mature GC anatomy. Given the potentially complex and conflicting interaction of directed chemotaxis with the dense packing of *cells*, we wondered how individual *cells* would move in the simulation. To assess this, we performed in silico experiments that replicated the in vivo intra-vital imaging studies from three separate groups. We generated a three-dimensional imaging window depicting a slice through the GC, and a subset of *lymphocytes* were tagged with a virtual dye, allowing them to be tracked over time. For each experiment we adjusted the dimensions of the imaging window, the time step, the minimum track length and the relative instantaneous velocity (see Methods) to match those of each group [9], [10], [18].

**Figure 7:**
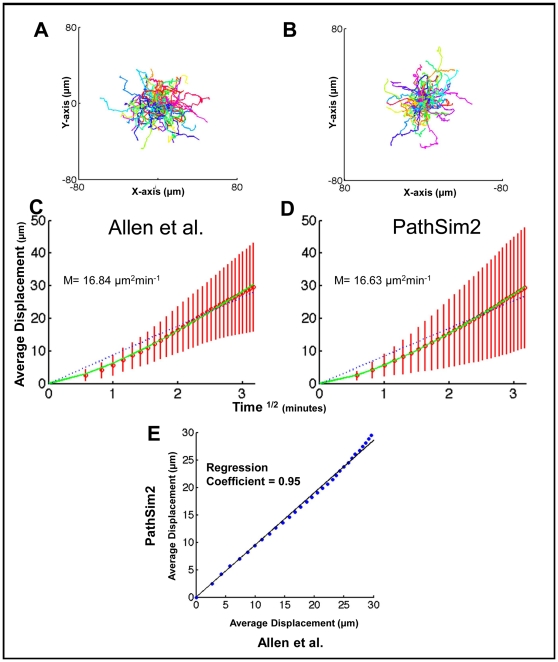
Random walk analysis of experimental (Allen et al.) and simulation output. (**A–B**) 10 min trajectory of representative tracks from (n = 100) from (A) Allen et al. and (B) PathSim2. (**C**) In vivo data from Allen et al. (n = 400; M = 16.84 µm^2^min^−1^). (**D**) PathSim2 data (n = 3741; M = 16.63 µm^2^min^−1^). The green line is the best fit regression line to the data points (red-bars, SD). Note the initial super linear behavior reflecting directed movement. The blue dashed line is the predicted best-fit for true random walk. At later times observed behavior approximates true random walk (linear over time^1/2^). (**E**) A linear regression between experimental and simulation output. The average displacement over time^1/2^ from the Allen et al. data set is plotted against the average displacement over time^1/2^ from the simulation data. A linear regression analysis (in the form of y = b*x) yields a regression coefficient (b) of 0.9544 (95% confidence intervals: 0.9459, 0.9628), indicating that these data are indeed comparable.

Figure 7 A-B shows the overall 10 min trajectory of individual GC B-cells from the Allen et al. data set compared to the simulation run under identical conditions. Figure 7C-D shows the average displacement of the cells versus time^1/2^, where a straight line is consistent with random walk [26], [27]. Over short time-scales (0–1.5 min^1/2^), both data sets show signs of persistent motion, as indicated by a super-linear increase in displacement versus time^1/2^ (as expected, this approaches linear when plotted against time (not shown)). Over longer time scales (1.5–3 min^1/2^), displacement becomes linear versus time^1/2^, consistent with cells undergoing random walk. This is the characteristic behavior that has consistently been observed in vivo [9], [10], [17] and has been referred to as PRW [17]. Given that none of this behavior was programmed, the similarity between in vivo data and simulation output was striking. The motility coefficients (M) were virtually identical, 16.8 µm^2^min^−1^ for the in vivo data set and 16.6 µm^2^min^−1^ for the simulation, and when the data were plotted against each other the resulting graph was a straight line with a linear regression coefficient of 0.95 (Figure 7E). We performed the same analysis comparing simulation output to the in vivo data from Schwickert et al. and Hauser et al. with the same outcome: simulation output closely replicated in vivo measurements (Figures S2 and S3). The simulation predicts, therefore, that the variations seen in the experimental data between the three groups can all be explained by technical differences in how the data were gathered and processed.

There were two potentially trivial explanations for the PRW we observe in the simulation. First, the timing of the persistence phase (∼2 min) is very similar to the value we have programmed for the time it takes for a cell to re-orient itself in response to a new *chemokine* gradient (∼1–2 min). The value for this parameter is based upon experimentally observed behavior [28], [29]. However, when we varied its value over a wide range (5sec – 5 min), it had no significant impact on the observed persistence time (not shown). Similarly, the stochastic nature of *chemokine* production by the *FDCs* in our simulation (see Methods) could generate random fluctuations in the *chemokine* gradient that might account for the random re-orientation of the cells over time. However, when *chemokine* production was artificially fixed in the simulation to be produced at a steady rate, PRW behavior was still observed (not shown). We conclude, therefore, that PRW is an emergent property of the simulation.

### Densely Packed Environment and Competition for Space are Required for PRW

**Figure 8:**
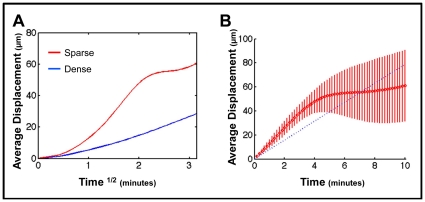
Dense environment is required for PRW. (**A**) Average cumulative displacement over time^1/2^ is shown for *GC B-cell* movement in a dense GC (blue ≡ in vivo, n = 5322), as compared to a sparse GC (red, n = 654) in which lymphocyte density has been reduced to ∼1/10 physiological values. Linear regression analysis comparing sparse to dense results in a regression coefficient of 2.5071 (95% confidence intervals: 2.4107, 2.6035). (**B**) Directed movement analysis of *GC B-cells'* movement behavior within the sparse environment from (A). Note that this analysis plots average cumulative displacement (shown in red, bars denote SD) over linear time. The blue dashed line is the best-fit regression for directed movement over the entire 10 min period. The linear increase in displacement over the first 4–5 min indicates directed movement.

Our simulation contains two components that modulate directed chemotaxis to produce correct GC anatomy. These are the densely populated environment and the consequent competition for space. We have performed control in silico experiments to test if both components are also required to produce a PRW from directed chemotaxis. In the first experiment, we adjusted the simulation to create a sparsely populated GC (∼1/10 physiological lymphocyte density). Under these conditions, *GC B-cells* do not follow a PRW trajectory, as seen from the average displacement when plotted against time^1/2^ (Figure 8A, regression coefficient of 2.51). Instead the *cells* tend to travel in a straight path for extended periods of time (∼4–5 min), resulting in displacement that is directly proportional to time (Figure 8B). This arises because there is no immediate impediment to directed chemotaxis.

**Figure 9:**
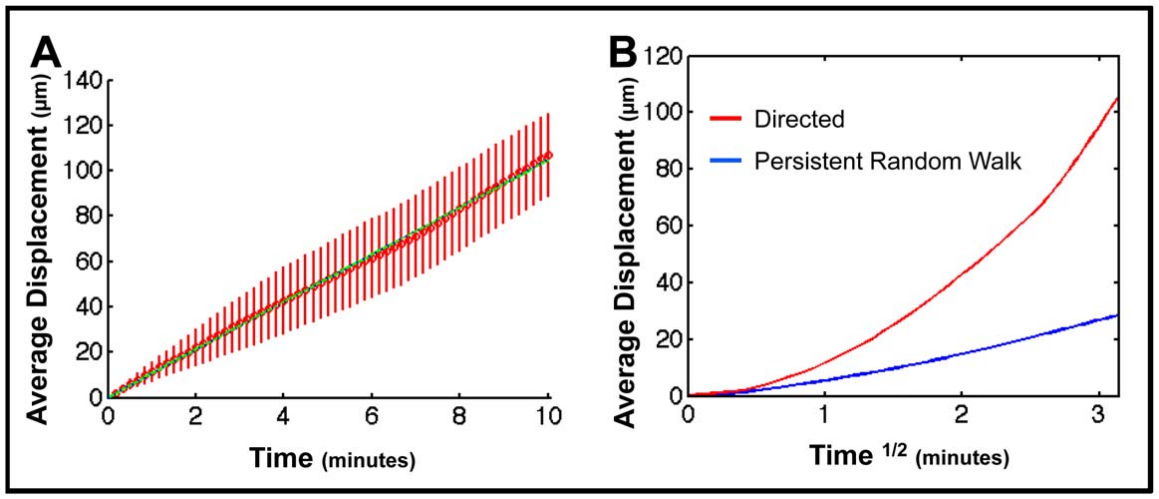
PRW emerges from chemotaxis-driven competition for space. (**A**) Directed movement analysis of *GC B-cells* (n = 105) moving through the extrafollicular region. Note that this analysis plots average cumulative displacement over linear time. The green line is the best fit regression line to the data points (red bars, SD). The blue dashed line is the best-fit regression for true linear movement. The green and blue lines are overlaid, indicating that this movement is entirely directed. (**B**) The directed (red) movement from (A) is shown in a random walk analysis, as compared to the PRW (blue ≡ in vivo, n = 5322) behavior of *GC B-cells* within the densely packed GC. Linear regression analysis comparing directed movement to PRW results in a regression coefficient of 3.2723 (95% confidence intervals: 3.1903, 3.3543).

In the second experiment, we tested the role of chemotaxis-driven competition for space. To achieve this, we used the simulation to artificially place a small number of *GC B-cells* in the extrafollicular *T-cell* zone. These *B-cells* are not competing with the *T-cells* for space, but instead move towards the follicle in a *chemokine* dependent manner. Analysis of this movement (Figure 9) demonstrated that it was directed (Figure 9A), with no suggestion of PRW (Figure 9B). This directed movement occurs despite the densely packed environment of the extrafollicular region. Thus, although a densely packed environment is required, it is not sufficient to modulate directed chemotaxis to produce PRW. This experiment is reminiscent of observed behavior in vivo, where antigen activated naive B-cells switch to directed movement as their chemokine preference changes [4], [5]. We conclude that both the densely packed environment and competition for space are required to modulate directed chemotaxis to produce a PRW.

Our simulation was designed to produce anatomically accurate GCs. This did not involve programming single *cells* to perform any other behavior than directed chemotaxis. Therefore, from this analysis we may conclude that the PRW behavior of *GC B-cells* in the simulation is a fully emergent property. To our knowledge, this is the first in silico model to accurately generate PRW as an emergent behavior of *GC B-cells* while retaining the anatomically correct architecture of the GC.

### *GC B-cell* movement recapitulates in vivo dynamics

**Figure 10:**
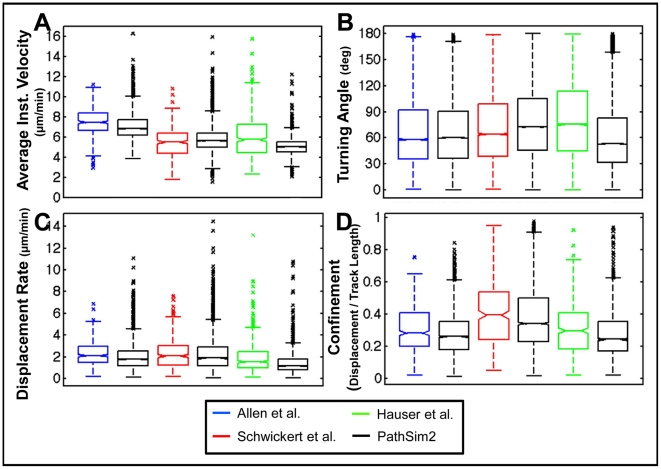
GC-cell movement behavior. Motility parameters of simulation *GC B-cells* (n = 1407-3741) compared to in vivo measurements of GC B-cells from Allen et al. (n = 400), Schwickert et al. (n = 310) and Hauser et al. (n = 483). Data are from a 30 min (Allen et al., Schwickert et al.) or 60 min (Hauser et al.) imaging session. Simulation data are acquired with comparable imaging parameters to the matched in vivo study, and should be contrasted to the in vivo data set to the left. Note that the observed velocity is always lower than the programmed velocity because movement is impeded in the densely packed environment of the GC.

We have demonstrated that a GC simulation capable of producing correct anatomy also predicts, as purely emergent behavior, the single cell movement phenomenon of PRW. We therefore sought to discover in what detail our simulation correctly predicted single cell movement dynamics in vivo by performing a comprehensive movement analysis on populations of individual *GC B-cells* compared to experimental data from three different groups [9], [10], [18]. We measured average instantaneous velocity, displacement rate, turning angle, and confinement [26], [27]. As before, simulation conditions were created to match the experimental conditions used by each group. In every case, the simulation output qualitatively replicated the experimental data from all three studies (Figure 10). There were only minor discrepancies with the velocity and turning angle measurements from the Hauser et al. data set. The cause of this difference is unclear (see Discussion). These results demonstrate that, in addition to recapitulating PRW behavior, our model is able to broadly reproduce the detailed cellular motility observed in vivo by three different groups.

### Inter-zonal crossing rates are consistent with cyclic re-entry GC models

**Figure 11:**
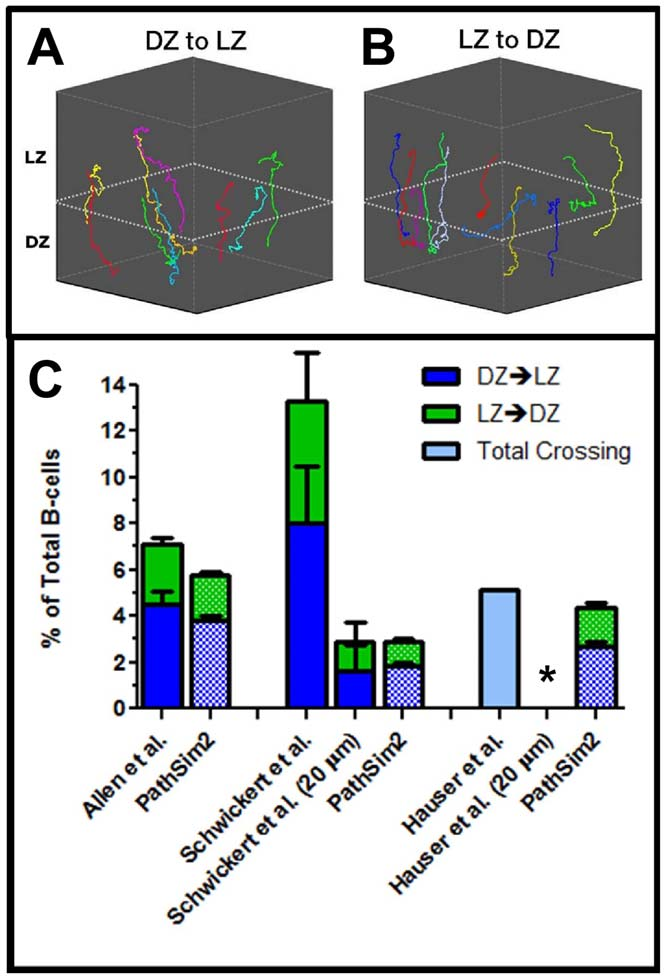
Inter-zonal Crossing. (**A–B**) Representative crossing tracks from the simulation over a 30 min imaging period. The dashed line approximates the LZ/DZ boundary and is included for reference. (**C**) Inter-zonal crossing frequency of in vivo and paired PathSim2 output. The crossing analysis of all in vivo data sets was previously determined by the original authors. We reanalyzed the data sets from Schwickert et al. and Hauser et al. applying a 20 µm boundary. The frequency of cells crossing between zones was measured during a 30 min (Allen et al., Schwickert et al.) or 60 min (Hauser et al.) imaging session (Allen et al., n = 698; Schwickert et al., n = 257; Hauser et al., n = 117; PathSim2, n = 3177-3528). Simulation data were acquired using an experimental setup identical to each matched in vivo study. The imaging windows used were Allen et al., 108 µm; Schwickert et al., 50 µm; Hauser et al., 30 µm. (Note that the error bars for original Schwickert et al. crossing frequencies were estimated from the published graph).

As discussed above, the standard model for GC dynamics is the cyclic re-entry model which predicts that cells cycle back and forth between functionally distinct DZ and LZs over the course of a cell cycle. For ease of discussion, we have referred to our default simulation as a modified cyclic re-entry model since we have added a modification based on experimental observation, namely that centroblasts occasionally remain in the LZ to undergo cell division [10], [18], [20], [21]. This modification was only added for biological accuracy and has little or no effect on cell dynamics (see below). Three groups have measured the rates of inter-zonal exchange [9], [10], [18], of which one reported rates too low to support a cyclic re-entry model. Instead they proposed an intra-zonal circulation model in which there is only limited exchange of cells between the zones [18]. We have addressed this issue by comparing our simulation to experimental output from all three groups. Representative predicted crossing tracks from the simulation over a 30 min period are shown in Figure 11A,B and display the expected directed trajectory of cells crossing between zones. In the simulation, predicted crossing tracks all represent true inter-zonal exchange because the simulation tracks the location of every *cell* and can distinguish local movement from true crossing. Note that, just as in vivo [10], the cells switch from PRW to directed movement when their *chemokine* preference changes. This is a further demonstration that PRW is an emergent, not a pre-programmed, behavior in our simulation.

**Figure 12:**
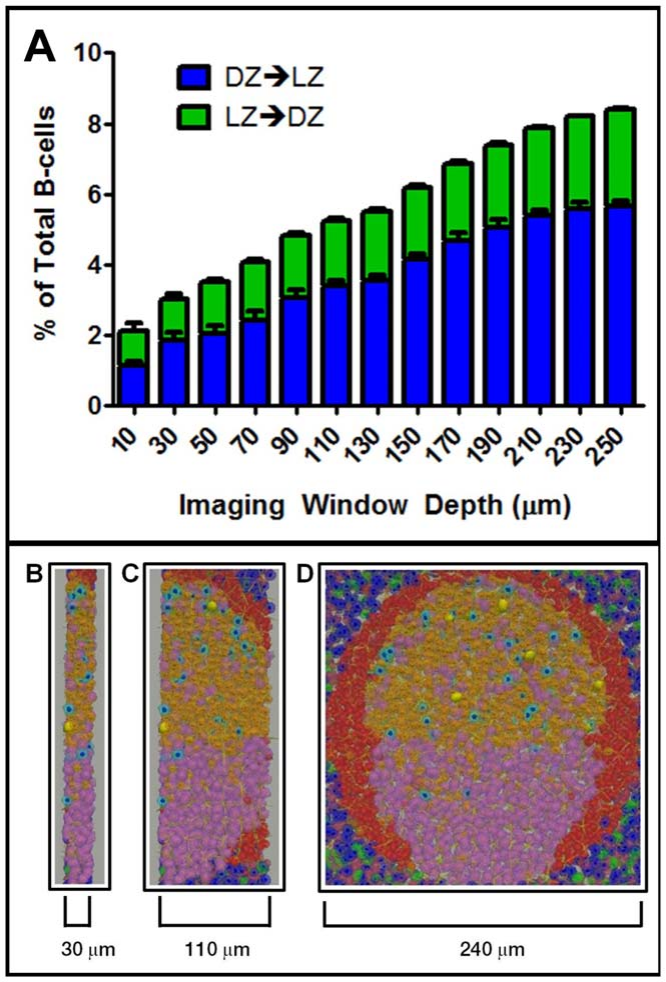
The effect of varying imaging window depth on inter-zonal crossing measurements. (**A**) The crossing frequency of *GC B-cells* (n = 1297-5805) was assessed over varying imaging window depths during a 30 min imaging session. Each analysis was performed in triplicate from independent GC simulations and error bars indicate SEM. (**B-D**) Visualization depicting the relative thickness of various window depths, representing windows used by Hauser et al. (B) and Allen et al. (C). The entire GC is shown in (D). In all frames, *LZ cells* in the process of crossing from LZ→DZ are shown in yellow.

In Figure 11C we compare predicted simulation output for zonal crossing with the results from the three experimental groups:

- Allen et al. required that cells cross a boundary of ∼20 µm between the LZ and DZ (NB: this is an inter-zonal boundary and is distinct from the cross-sectional dimensions of the imaging window). When we applied their conditions to the simulation it correctly predicted results consistent with the experimental data (Figure 11C). Thus, the inter-zonal crossing frequency reported by Allen et al. is consistent with our modified cyclic re-entry simulation model.
- Schwickert et al. used a less stringent 0 µm inter-zonal boundary. When we applied their experimental conditions to the simulation, we predicted a significantly lower crossing frequency than was reported (Figure 11C). We hypothesized that this discrepancy arose because the 0 µm boundary cannot always distinguish random movement across the boundary, generated by the high motility of GC B-cells, from true crossing, and therefore will tend to overestimate this value. To test this hypothesis, we re-analyzed the original experimental data set (kindly supplied by the investigators) applying a 20 µm boundary. As predicted, most previously identified crossing tracks failed this test and we observed an inter-zonal crossing frequency virtually identical to that predicted by the simulation. Therefore, this data set is also compatible with our modified cyclic re-entry simulation model.
- Hauser et al also employed a 0 µm inter-zonal boundary. When we applied their experimental conditions to the simulation, the predicted inter-zonal crossing was compatible with the experimental data (Figure 11C). (NB: their imaging session was twice as long, 60 min, as the other two and they did not report directionality, only total crossing). However, when we re-analyzed this data set (kindly supplied by the investigators) applying a 20 µm boundary, we observed no crossing tracks. We have used the simulation to explore this observation. We hypothesized that it was related to the size of the imaging window used. The simulation predicts that the number of crossing events observed per unit area will decrease as the imaging window deceases (Figure 12). This is because the track must remain within the imaging window for a minimum amount of time to be included in the analysis, and the likelihood of this for any given track decreases with the imaging window thickness. We have used simulation graphics to demonstrate that Hauser et al. employed the narrowest window (Figure 12B), which would have significantly impaired their ability to detect crossing. A second contributing factor is error introduced during track reconstruction. The dashed lines in Figure S4 display the predicted distribution, from the simulation, of track lengths for the various imaging window dimensions used. As expected from the above discussion, narrower imaging windows under represent longer track lengths (the ones most likely to cross a 20 µm boundary). However, when we compared the predicted to the experimentally observed track lengths, the later even further under represented long track lengths. We assume that this represents intrinsic error associated with track reconstruction, mainly the difficulty in successfully following a single cell over time. We propose that the absence of observed crossing, when applying the 20 µm boundary to the Hauser et al. data set, is a consequence of the intrinsic error associated with track reconstruction and the narrow imaging window, both of which militate against the detection of longer track lengths.

### Cyclic re-entry models are robust

The major objection to cyclic re-entry models was based on the frequency and interpretation of inter-zonal crossing rates. Therefore, we used the simulation to test the robustness of our model by varying two parameters that might be expected to affect crossing rates sufficiently to confound it.

- Division in the LZ: As noted above, we have added a modification to the original cyclic re-entry model based on experimental observation, namely that centroblasts occasionally remain in the LZ to undergo cell division [10], [18], [20], [21]. We estimated this from published literature to be ∼20%. We have varied this value in the simulation from 0% (as described in the original cyclic re-entry model) to 40%, well above what has actually been observed (Figure S5A). As expected, the crossing frequency decreases as the rate of LZ division increases; however, the changes were minimal and remain compatible with experimental values.
- Cell cycle time: Reports of cell cycle time for GC B-cells have ranged from an average of ∼8 hr (Hauser et al.) to ∼12 hr or longer (Allen et al.). The default value for our simulation is ∼10 hr. We used the simulation to predict the result of varying the cell cycle time from 6 hr to 14 hr. As the average cell cycle time increases, the number of *GC B-cells* observed crossing between zones decreases (Figure S5B), as expected. However, these differences were minimal across the range of times tested and remain compatible with published experimental values.

This analysis demonstrates that our model is not compromised when tested across the range of experimentally credible values, indicating that it is robust and stable. We conclude, therefore, that inter-zonal crossing rates are compatible with all the variants of the cyclic re-entry model that we have tested, including the original and our modified version.

## Discussion

In this study we have recapitulated the anatomy of the primary follicle, the expansion phase of the GC and the mature GC (including correctly structured MZ, DZ and LZ), based solely on chemotaxis. We have gone on to show that correct single cell movement dynamics, compatible with published experimental observations, arise from this simulation as purely emergent properties. However, chemotaxis can only achieve this when constrained by the known biological properties that cells are incompressible (i.e., in response to external forces, *cells* can change shape but their total volume remains constant) and exist in a densely packed environment, and must therefore compete for space. It is this interplay of chemotaxis and competition for space that generates all the complex and biologically accurate behaviors described here. Thus, in addition to competition for antigen and T-cell help, we have identified an additional critical force driving GC development, namely chemotaxis driven competition for limited space. We believe that this is the first GC model that is able to recapitulate both correctly detailed anatomy and single cell movement with a single simple mechanism that is well documented in the biological literature.

Our simulation models lymphoid tissue, therefore we have used directed chemotaxis as the biological mechanism that drives movement. However, during the course of our investigations it became apparent that the key property in shaping anatomy is relative *cell* velocity. That is to say, if two populations are moving at different speeds while competing for space, high level structure will evolve whereby the slower species is excluded. Such a distinction in velocity could arise through differential responses to a chemokine and/or through variable strength of binding/adhesion to stroma that preferentially facilitates the movement of one population. It was not self-evident to us that this alone would be sufficient to delineate sharp tissue boundaries, and we believe it has important implications for other biological and physical systems where sharp boundaries are generated without a physical barrier. In our case, this is most eloquently demonstrated in the expansion phase of the GC, where changes in the *FDC* network cause *naive B-cells* to move more slowly than *GC B-cells*. This alone is sufficient to allow the *GC B-cells* to drive the *naive B-cells* out of the follicle and create a sharply defined MZ. The requirement for dense packing then becomes self-evident because one cell population cannot drive another out unless space is limited.

Perhaps even more surprising to us was that these same simple properties of our model were sufficient to produce, as purely emergent behavior, correct single cell movement dynamics including PRW, the full range of commonly measured motility parameters and inter-zonal crossing. This was both unexpected and unplanned in our studies, and the accuracy with which our simulation recapitulated this movement speaks to the power of this observation. The crucial role of the environment in modulating chemotaxis to produce this movement is most clearly demonstrated in our control simulations where a *cell* not competing for space (i.e., in a sparsely populated environment or when headed elsewhere by directed chemotaxis), simply passes through the space. That is to say, unless a *cells'* movement is modulated by competition for space, it will follow the biologically accurate behavior it is programmed to do and migrate towards the *chemokine* gradient to which it is responding.

Recently, two groups have also used modeling to study the movement behavior of cells in lymph nodes [17], [19]. Neither of those studies was able to produce correct anatomy and single cell behavior as emergent from a single, simple model of individual cell movement. Beltman et al. were the first to suggest a role for the densely populated environment in modulating lymphocyte movement [19]. They propose a model of individual cell movement in which a cell attempts to move in a preferred direction for a defined persistence time. This persistence time was selected empirically to produce desired movement behavior; there was no biological basis for its value and the model was extremely sensitive to small changes in this parameter. They observe that in a densely populated environment, a *cell* will often be unable to proceed in the preferred direction and will change to the direction where it is able to move, i.e., the preferred direction is not driven by a chemokine gradient. Using a computer simulation, they demonstrate that this model results in observed behavior that matches the PRW of in vivo experimental data. Both their model and our model have persistence time as a component, but in our model persistence time is an emergent property of the model, not a programmed behavior.

The Beltman et al. model cannot be used to explain inter-zonal migration or the formation and maintenance of correct anatomy. Indeed, in their model *cells* are artificially confined to a packed environment, otherwise they will simply diffuse away, whereas our model actually generates a densely packed environment (e.g., in the follicle) without physical constraint, thus, correctly recapitulating biological behavior. This is because *cells* of the same type are all following the same (or similar) *chemokine* gradients. For example, if we uniformly and sparsely populate simulated lymphoid tissue with *activated B-cells* and have a single follicle in the center of that tissue, all of the *activated B-cells* will move into that follicular region and create a densely populated environment in which PRW will emerge. This generation (and maintenance) of a packed environment does not occur in the Beltman et al model.

In the study by Figge et al. they concluded that directed chemotaxis was not sufficient to explain the origins of GC structure or PRW [17]. They suggested that to recapitulate in vivo dynamics it was necessary for *cells* to go through periods when chemotactic responses were weakened or absent, during which time an individual *cell* randomly selects the direction in which it moves. Thus, in their study, they assumed that PRW was an intrinsic (and therefore pre-programmed) property of the individual *cells,* rather than an emergent behavior as in Beltman [19] and in our model. When “strong” chemotaxis was then applied, it overrode the *cell's* PRW movement component and produced directed movement, allowing cells to move towards a *chemokine* source. However, in order to both maintain GC structure and allow the re-expression of PRW, it was then necessary to significantly weaken or turn off chemotaxis. As a consequence, the observed behaviors were highly sensitive to the value of the chemotaxis parameter, and they noted the contradiction that their model actually broke down under physiological conditions of a densely packed environment. (We note that their simulation framework was not designed to handle a densely packed environment. If they had designed their simulation framework with this feature, we suggest that the pre-programmed PRW component in their model would be unnecessary because it will emerge when cells are moving in the densely packed environment.) From our studies it is apparent that there is no need to invoke new mechanisms to reconcile conflicts between observed movement behavior and chemotaxis. Indeed, our simulation predicts that correct movement behavior is, in fact, an emergent property of chemotaxis itself when modulated by competition in a crowded environment. As a consequence, our model is relatively insensitive to variations in the chemotaxis parameter and performs correctly, with a physiologically densely populated environment. Indeed the densely populated environment is itself an essential, emergent property of our simulation. Therefore, our interpretation of the Figge et al. study is that PRW cannot be an intrinsic property of GC B-cells because it produces irreconcilable conflicts with directed chemotaxis.

Another important contribution of our study is to show that the published experimental data on inter-zonal crossing rates from two separate laboratories are as predicted by our model. In our simulation, all of the predicted crossings represent true inter-zonal exchange because the simulation tracks the location of every *cell* and can distinguish local movement from true crossing. Experimentally, we believe that the stringent crossing method of Allen et al. is more reliable because it requires cells to traverse a clearly defined 20 µm boundary between zones. In comparison, the less restrictive 0 µm boundary is subject to an increased amount of false positives. Without the use of a boundary, it is difficult to distinguish true inter-zonal crossing from tracks that simply meander back and forth across the estimated LZ/DZ interface as the result of random local movement. Consequently, only when we re-analyzed the Schwickert et al. cell tracks applying a 20 µm boundary, did we find inter-zonal crossing rates comparable to those predicted by the simulation. Our model is a version of the cyclic re-entry model that was modified to allow the observation that significant cell division is seen in the LZ. As already discussed, this modification was included for biological accuracy and did not have a significant impact on the behavior of the model. Therefore, we can conclude that the experimental crossing data are consistent with all of the cyclic re-entry models we have tested and there is no need to invoke other models. Indeed, confirmation of cyclic re-entry dynamics in vivo was provided by a recent follow up study from the Schwickert et al. research group [20], where they determined inter-zonal exchange using new, more quantitatively accurate methods.

Concern over the validity of the cyclic re-entry model arose because Hauser et al. reported crossing rates that they believed were too low to accommodate such a model. Indeed, when we applied a 20 µm boundary to their data set we did not detect crossing. When we interrogated this result with the simulation it became apparent that it may have been due to technical issues, notably the very narrow imaging window used. From our analysis, the simulation predicts that a cyclic re-entry model would require that there should only be ≤5 cell crossing events in this data set, and this low number of crossing events may have been missed. There are several possible reasons for this. First, a narrow imaging window is more difficult to align correctly and reduces the likelihood of a crossing track remaining within the window while crossing the 20 µm boundary zone (see Figure 12 B-D). In addition, we have shown that the experimental data from all three groups is intrinsically biased against recording the longer tracks which are the ones most likely to cross the boundary (presumably for technical reasons related to track reconstruction, etc). Lastly, a reduction in the percentage of tracks identified as crossing occurs because, over the course of an imaging session, a single cell may exit and re-enter the window. Our simulation demonstrates that this becomes exacerbated as the imaging window becomes narrower (track/*cell* ratio ∼1.15 for a 30 µm window, ∼1.10 for a 50 µm window and ∼1.05 for a 108 µm window). Thus, the total number of apparent tracks observed will increase as the window becomes narrower, causing the percentage of crossing tracks per total tracks to decrease. Because of these considerations, we conclude that the Hauser et al data set is not incompatible with cyclic re-entry models.

To obtain good correlations between predicted and observed cell movement behavior, it was important to match simulation and experimental conditions for each experimental group. This strongly implicates technical variation in explaining differences in the data sets. Our studies indicated that differences in the time step and instantaneous velocity contributed most to variation between the three groups when addressing singe cell movement (not shown). Imaging window size only became crucial when measuring inter-zonal crossing rates. It was surprising to us that when normalizing the three in vivo data sets to common experimental conditions (i.e., imaging window dimensions, sampling time and the minimum track length) we found that they differed slightly in the observed velocity of GC B-cells (Figure S6). It is not entirely clear why this should occur, but may reflect on individual experimental conditions (i.e., preparation of imaged lymph node, microscope resolution, transgenic GC B-cell antigen affinity, mouse model, etc.). Overall, though, it was striking that the movement behavior predicted by our model matched the experimental observations from three separate experimental groups so precisely. This is a strong endorsement of the quality and validity of the experimental studies, as well as the utility and robustness of our simulation.

Our model does not explicitly include all the details of lymphocyte movement observed in vivo. For example, Bajenoff et al. has demonstrated that B- and T-cells move throughout lymphatic tissue in close association with stromal networks (FDC, FRC), and cell turns correspond with branch points in the network [30]. They concluded that observed behavior (PRW) is a consequence of movement down a randomly structured stromal network. However, we were able to generate similar dynamics without the explicit inclusion of migration on the *stromal* network. While stromal cells may serve as tracks on which lymphocytes travel, we suspect that branch points alone do not account for the observed movement behavior, as cells don't always turn at each stromal network intersection. The resolution of this apparent conflict lies in the fact that within lymphatic tissue the stroma extends out in a dense three-dimensional network, providing the potential for a cell to travel in a full range of directions at all times, and that chemokine cues guide the actual direction of motion and turning behavior.

In conclusion, we have constructed a GC simulation which, for the first time, successfully recapitulates both large scale anatomy and small scale single cell dynamics. This was achieved using only fundamental established biological properties of directed chemotaxis, competition for space, and a densely populated environment. We believe that the ability to generate sharply delineated tissue structures applying only these principles may have widespread relevance in the fields of cellular and developmental biology.

## Methods

### PathSim2 Overview

PathSim2 is a general software framework for the simulation of lymphatic tissues at the cellular level (NB: the simulation framework for the GC model described here is available for download as a supplemental program written in C++). It is an agent-based simulation that renders a small piece of tonsil lymphatic tissue, termed the Basic Tonsil Unit (BTU). The BTU consists of a single follicle and all the relevant surrounding tissue necessary for its function (Figure 1, Video S1). With the BTU, we have developed a model of GC development based on known biological parameters (listed in Table S1). The framework simulates lymphocytes as discrete agents that are able to move and interact with each other and their environment over time in a three-dimensional volume. This volume is discretized using a computational mesh and then partitioned into individual elements corresponding to this mesh. Individual agents have volumes characteristic of the cells that they represent. At every point in time, an individual agent has a position in the mesh, where that position is not restricted to a specific set of discrete points (i.e., not a lattice model). The framework ensures that the sum of the volumes of the agents contained within each element does not exceed the actual element volume (i.e., the total space available). This careful representation of space within elements is important to ensure physiological cellular density (see below and Table S1). Space within tissue can be occupied by mobile agents (e.g., *B-cells*), immobile agents (e.g., *FDCs*) and inert agents; in the presence of a *chemokine* gradient, the *cell* with the best chemotaxis will preferentially occupy that space. Inert agents represent vascular or lymphatic fluids that occupy volume and are able to move throughout the tissue as well as being displaced by mobile *cells*. Based on our own estimates of the packing density of lymphocytes in tonsil histology sections, the displacement of inert agents is restricted to ensure that they occupy a minimum of 15% of the space within an element and, therefore, is an approximation. Using similar approximations, immobile agents (*stromal cells*) occupy 25% of the space, leaving the remaining ∼60% of space for mobile agents (*lymphocytes*); this is our estimate of physiological lymphocyte density and our definition of a densely packed environment. The validation of these approximations comes from the result that we achieve the correct size germinal center, with the expected number of cells, given published values of cell sizes. In the case of the sparsely packed GC, *lymphocyte* density is reduced to ∼1/10 of the default value and held at this level; this is accompanied by an equivalent increase in inert agent density. This sparsely-packed condition was generated to demonstrate the effect that *lymphocyte* density had on GC morphology and cellular movement behavior. This does not accurately model conditions in vivo where lymphocyte density has been lowered either artificially or genetically.

Within PathSim2, individual agents have unique internal states that allow them to undergo a variety of state transitions based on a number of factors. These factors include time in state and external factors such as interactions with other agents and local *chemokine* concentrations. These state transitions are specified as parameters that are input to PathSim2. For example, the transitions illustrated in the model given in Figure 3B are implemented using this facility. Akin to a cell's differentiation state, an agent's state determines how it will respond to its environment and other agents. Agent behavior is further influenced by the dynamic expression of signaling proteins. Agents are capable of expressing signaling proteins both internally and externally, the latter bound to the surface membrane. The accumulation of internal proteins can lead to specific transitions when a pre-determined threshold is reached. Internal protein levels are regulated over time and in response to interactions with the environment and other agents. External membrane bound proteins allow for a direct information exchange during physical interactions between agents. Individual agents can interact with other agents based on proximity, probabilities of interaction, and the agents' internal states. Again, these parameters are specified as part of the model input to PathSim2. A summary of these parameters is given in Table S1.

As a whole, PathSim2 reconstitutes overall human immune system dynamics, allowing us to efficiently simulate physiological lymphocyte populations in an average human (∼500×10^9^). To achieve this, the software framework includes an implementation of “pools” which are compartments that, unlike the tissue, contain agents but without specific positions. For example, the blood and lymph compartments are represented as pools. Agents can flow to and from these compartments into the tissue based on sources and sinks that are programmed by the model to be located in particular tissue types. Flow rates between source and sink compartments are determined by the model parameters, but individual *cells* may be more (or less) likely to move between compartments based on the relative *chemokine* concentrations present in these source and sink compartments. The pool dynamics are not a significant aspect of the computational results presented in this paper other than as the source of *naive lymphocytes* and as sinks to the lymph pool. The flow between these sources and sinks does result in an overall “global” flow from tissues with sources to tissues with sinks. However, it is important to note that the speeds associated with this global flow are small (by an order of magnitude or more) relative to agent chemotaxis speeds. As a result, we believe that this flow rate does not significantly affect the movement statistics presented in this paper.

### A modified cyclic re-entry GC model

In the BTU the epithelial layer is in contact with the saliva and is involved in the transport of antigen to the underlying lymphoid tissue. We have chosen to simulate a human tonsil, but at our current level of detail the tissue architecture within the BTU could also represent a typical peripheral lymph node, where afferent lymphatics would replace saliva as the source of antigen. Beneath the epithelium are high endothelial venules (HEVs) from which *B-cells* and *T-cells* emerge from the peripheral circulation (reviewed in [31]). The follicle and extrafollicular region contain stromal networks consisting of *follicular dendritic cells* (*FDCs*) and *fibroblastic reticular cells* (*FRCs*) respectively (reviewed in [32]). *Lymphocytes* become localized within the BTU in response to *chemokine* gradients known to be produced by these networks. *FDCs* produce *CXCL13* (Figure 2B), towards which *naive B-cells* migrate (via CXCR5), and *FRCs* produce *CCL21/19* (Figure 2C), which attracts *naive T-cells* (via CCR7) (reviewed in [3]). *Lymphocytes* remain responsive to their respective *chemokines* for 12–24 hours [33]. At this point, they switch *chemokine* preference and begin to follow the exit *chemokine* gradient (*S1P*) generated by the efferent lymphatic vessels (reviewed in [33]). *Lymphocytes* then exit the tissue via the efferent lymphatics, which drain to the lymph system at the bottom of the mesh. The result is a BTU with a naive primary follicle at homeostasis (see Figure 2A).

The GCR is initiated by seeding 3 *GC founder B-cells* into the *FDC* network which begin to proliferate with a cell cycle time of ∼6 hr (Figure 4A). These *GC founder B-cells* proliferate until they receive a signal to differentiate into *centrocytes*, marking the end of the expansion phase and the beginning of the next, competitive phase of the GCR. In our model, this signal is given between hour 69–75 to allow a smooth transition into the next phase at around day 3, reproducing observed GC kinetics.

The default status of our simulation is that of a modified cyclic re-entry model. These models are characterized by the movement of GC B-cells between functionally distinct DZs and LZs (Figure 3B,5) driven by regulated gradients of zonal chemokines (Figure 5B,D). During the competitive phase of the GCR, the average cell-cycle time for a *GC B-cell* is ∼10 hr. *Centroblast* division takes ∼5 hr, producing two daughter cells that differentiate into functional *centrocytes* which change their *chemokine* preference to the *CXCL13* gradient generated in the LZ, towards which they migrate (Figure 3B).

In the LZ, *centrocytes* undergo positive and negative selection. They remain there for ∼5 hr, representing time spent interacting with antigen and competing for T-cell help. Positive selection is modeled as a competition for a limited amount of antigen that is continually presented by *LZ FDCs*. *Centrocytes* that lose this competition die by apoptosis (negative selection). This encapsulation is designed to result in a stable GC that fills the follicle and remains at equilibrium. Our simulation was developed to study the movement dynamics of *lymphocytes*; therefore, we do not specifically model antigen selection or affinity maturation. Positively selected *centrocytes* represent those cells that were able to successfully interact with antigen and receive the necessary amount of T-cell help. Of the positively selected *centrocytes*, the majority differentiate back into *centroblasts* (p_CB_), while the remainder leave the GC as output (*memory B-cells* or *plasma cells*) (1-p_CB_). Most *centroblasts* then change *chemokine* preference and migrate back to the DZ (1-p_LZ_) pursuing *CXCL12,* the *chemokine* produced by activated *DZ stromal cells* (CXCL12^+^) [25], where they again initiate cell division. Our modified version also allows a fraction of centroblasts to remain in the LZ (p_LZ_) and divide there. This modification was added for biological accuracy and has little, if any, impact on GC dynamics (see Results). Note that the ratio of *centrocytes* to *centroblasts* that we achieve (∼1.6∶1, Figure 3A) is consistent with the originally published observations that centrocytes outnumber centroblasts at the peak of the GCR [24]. However, with slight modifications to the model (i.e., number of divisions by *centroblasts* in the DZ, length of positive selection of *centrocytes* in LZ) it is possible to reproduce the ratio of ∼1∶1.9 that has been observed in more recent, quantitative studies [20].

The GC is maintained at homeostasis by a steadily secreted *antigen*. Free *antigen* triggers the activation of the *FDC* network. Free *antigen* also enables *LZ FDCs* to present limited antigen to *centrocytes* (positive selection), and drives the production of activated *follicular T-helper* cells. While this current GC model does not require direct *follicular T-helper* interactions during positive selection in the LZ, *follicular T-helper* are present and occupy space within the LZ of the GC. *Follicular T-helper* activation is constructed to maintain a stable population in the GC (∼5% of total *GC-cells*) while *antigen* is present.

### Chemokines

*Lymphocytes'* movement within the BTU is only driven by directed chemotaxis. We have modeled chemotaxis based strictly on the known mechanisms and movement of lymphocytes in response to chemokines (reviewed in [34], [35], [36]). Chemokines are represented as scalar concentrations at the element centers. In the mesh, *chemokine* concentrations at any location can be determined by interpolation from concentrations in nearby elements. The movement of *chemokines* in our simulation is regulated by Brownian motion derived from first principles based on the solution of the time-dependent diffusion equation. The *chemokine* diffusion coefficient is estimated from the effective size of a chemokine molecule and the viscosity of the tissue fluid (for which the viscosity of water is used) [37].

The production of *chemokine*s is regulated by a negative feedback mechanism. *Chemokine* is produced until a threshold concentration is reached within each element. At this point, further *chemokine* secretion ceases until the local concentration drops below the threshold level. This mechanism results in the unsynchronized release of *chemokine* by individual *FDCs* throughout the follicle. Therefore, from a *B-cells'* perspective within the follicle, the local *chemokine* concentration will remain within a range, but will fluctuate due to the feedback mechanism as well as the consumption of *chemokines* by other agents. Outside of the follicle, the concentration of *chemokine* sharply declines. This model was implemented to generate *chemokine* gradients that are compatible with biological data, in which the chemokine concentration is relatively uniform throughout the follicle. Note that we have also examined a model in which *chemokine* production by *FDCs* is continuous. Under these conditions, PRW behavior still emerges (data not shown). We can conclude, therefore, that PRW behavior is not a direct result of small fluctuations in *chemokine* concentration in the follicle.

In our simulation, there are multiple factors besides diffusion that affect a *chemokine's* concentration and help shape the gradient. The model includes sources and sinks for each *chemokine*. Sinks include *chemokine* internalization by responsive *lymphocytes* and non-specific degradation. The rate of internalization (“consumption”) by responding *lymphocytes* is a simulation parameter and scales with *cell* size. As a consequence, loss of total *cells* under sparsely-packed conditions will result in increased *chemokine* levels. However, while the magnitude may change, the shapes of the *chemokine* gradients are relatively unaffected (Figure S7). Non-specific degradation is also a simulation parameter and encapsulates potential chemokine loss from destruction by proteases, as well as adhesion to components of the stromal network. Most importantly, this innate degradation is required to ensure that the *chemokine* concentration drops off outside of the tissue where it is actively being produced (e.g., the decline of the C*XCL13* gradient in the extrafollicular zone). Ultimately, our goal was to generate appropriately shaped *chemokine* gradients throughout the BTU that replicate the estimated physiological levels in vivo [4], [25], [38] and are within the known optimal range of lymphocyte responsiveness [39]. Our interest was examining *lymphocyte* movement towards *chemokine* gradients within densely packed lymphoid tissue not the detailed mechanism that generates the gradient. There is supportive biological evidence that these gradients exist in vivo, and we know that responsive lymphocytes will follow these gradients. Thus, the critical level of detail for our model is the *chemokine* gradient and the behavior of the *cells* responding to it. How this gradient is produced and maintained is not germane to our study. Our encapsulation of the mechanism by which *stromal cells* generate *chemokine* gradients allows for the simplification of the biological system, as the interactions of the individual components of this pathway are not necessary to recapitulate known behavior. Thus, the encapsulations used in our explanatory model allow us to identify the mechanisms that are both necessary and sufficient.

Note that we report the concentration of free *chemokine*, which is a direct representation of the gradient visible to responsive *cells*. This value will be lower than in vivo measurements based on histology, protein quantification, and PCR, as it is difficult to distinguish the true amount of free chemokine in solution from that bound to the surface of lymphocytes/stromal cells with these methods. Based on the estimation that *lymphocytes* express roughly 10^2^–10^4^ chemokine receptors [5], [20], [25], [40], we developed a *chemokine* model that results in a maximum of ∼10^4^ bound *chemokine* receptors on a *lymphocyte.*

### Chemotaxis

Chemotaxis is modeled as an interaction between individual agents and the local concentrations of *chemokines* to which they respond. In the crowded environment of the lymph node, the levels of *chemokines* reaching a *lymphocyte* surface are affected by its position relative to the *chemokine* gradient (source and sinks), as well as competition for the *chemokine* by other responsive *lymphocytes* in the area. As the *lymphocyte* moves, bound *chemokines* are internalized while new *chemokine* molecules continue to accumulate on the surface. The target direction for an agent is determined from the location of the maximum *chemokine* concentration accumulated on the surface of the agent. If unimpeded, the *lymphocyte* always moves in this direction (Figure S1). The direction is re-computed at a time-interval corresponding to the published value for the time it takes a lymphocyte to re-orient in a chemokine gradient (∼1–2 min) [28], [29]. (Note: this parameter does not control the observed persistence time of lymphocytes undergoing PRW as discussed above.) In densely packed regions such as the GC, many agents may have the same target direction. However, all agents cannot move in this common direction because of their incompressibility. That is, there is a competition for space amongst agents. This problem was solved by the flow solver described below.

Briefly, the flow solver acts to enforce an incompressibility constraint during agent movement that occurs at each time step of the simulation. The incompressibility constraint ensures that no element within the tissue is allowed to contain more agents that can physically be accommodated in the volume of the element. This physical constraint arises because cells, being composed primarily of water, are not compressible – i.e., they can change shape due to external forces, but not volume. To enforce this constraint, the flow solver discretizes elements into sub-elements, where the volume of each sub-element is smaller than the volume of the smallest agent in the simulation. Agents are not constrained to discrete positions in the tissue, as is the case with a lattice model. Further, an agent's shape is not constrained to an aspect ratio of one, but instead may change due to external forces. At each time step, an agent has a direction it desires to move and a shape it attempts to take. The combined movement and shape changes of all agents will, in general, result in a violation of the incompressibility constraint. To enforce this constraint, we find a flow velocity over each sub-element that, when combined with the desired movement of each agent, will maintain the incompressibility constraint. This "flow velocity" is not a physical flow, but rather is intended to represent the forces that result when agents push against one another to move into the same space. To find the flow velocity, an incompressible flow problem is solved on the discretized tissue, where a potential flow variable is associated with each sub-element and a source (sink) is computed for each sub-element based on its current agent volume. Because it is coupled with the agent movement, the incompressible flow problem is nonlinear and, therefore, is solved iteratively. When the iterative process is complete, the movement of an agent is the sum of the agent's desired movement vector and the local flow velocities that act on that agent. The final movements result in new global agent positions/shapes that satisfy the incompressibility constraint.

In summary, this solution computes agent positions taking into account the motion of individual agents in their chemotaxis directions and the individual agent-agent “forces” resulting from the incompressibility condition. The key point of this solution method is that it allows for the chemotaxis of individual agents while maintaining physically mandated incompressibility. In fact, an agent's ability to change aspect ratio (as depicted in Figure S1C) is the essential component of these algorithms that allows agents to efficiently move through the tissue and around other agents. As a result, we believe that the observed change in cell shape during chemotaxis facilitates these cells' motion in crowded tissue.

The simulation advances based on a specified time-step. For this model we have used a time-step of 10 sec, however we have observed similar dynamics over a range of time-steps (5–15 sec). At each time-step, the solution of the complex fluid flow problem is solved, and the results of the chemokine diffusion problems, agent-agent interactions, and agent state changes are determined.

### In Vivo Data and Tracking Parameters

Complete primary data sets were made available to us through the generosity of Drs. Christopher Allen, Takaharu Okada, and Jason Cyster (UCSF), Tanja Schwickert and Michel Nussenzweig (Rockefeller University), and Anja Hauser and Ann Haberman (Yale University).

Allen et al. used an imaging window of 240 µm x 288 µm (xy plane) with a depth of ∼108 µm and images were acquired every 20 sec for a 30 min time period. A cell track was required to remain within the imaging window for a minimum of 10 min. Cell tracks used in the motility analysis are derived from 5 imaging sessions (n = 400), while the crossing analysis is derived from an additional 6 imaging sessions (n = 698). The authors separate the LZ and DZ by an estimated ∼20 µm boundary.

Schwickert et al. used an imaging window of 300 µm x 300 µm with a depth of 50 µm and acquired images every 37 sec for a period of 30 min. Cell tracks were required to remain within the imaging window for a minimum of 2 min. Cell tracks used in the motility analysis are derived from 6 imaging sessions (n = 310), and the crossing analysis is comprised of 5 of these sessions (n = 257). The authors estimate the LZ/DZ boundary using a flat plane.

Hauser et al. used an imaging window of 312 µm x 312 µm with a depth of 33-44 µm (effectively sampling ∼30-40 µm) and images were acquired every 15 sec for a period of 60 min. Cell tracks were required to remain within the imaging window for a minimum of 5 min, unless they displaced greater than 20 µm. Cell tracks used in the motility analysis include all GC B-cells (originally classified as stationary and motile by the authors) and are derived from 3 imaging sessions (n = 483), while the crossing analysis was performed on motile cells (displacement greater than 15 µm) from 1 session (n = 117). The LZ/DZ boundary was estimated using a flat plane.

PathSim2 is capable of reproducing the dimensions of any imaging window, as well as analyzing the whole GC. For all of our analyses, a subset of *lymphocytes* were tagged with a virtual dye, allowing them to be tracked (∼35–50% of agents within a single GC; n = ∼1500–5000, depending on window depth and length of session). For comparisons with in vivo data the dimensions of the imaging window we used and the elapsed time interval between frames were adjusted to match those used in each experimental study. For example, when modeling the work of Allen et al., we used their window depth of 108 µm and tracked *B-cell* positions every 20 sec. By default, when not mimicking experimental conditions, we performed a whole GC analysis (without an imaging window) and tracked agents every 10 sec. The LZ/DZ boundary used in the crossing analysis was exact and defined by the location of *LZ FDCs* and *DZ stromal cells*.

For most simulations we generated a three-dimensional imaging window depicting a slice through the GC analogous to in vivo intra-vital imaging studies. However, we also took advantage of the simulation to measure motility parameters in the whole GC in comparison to those observed within the constraints of an imaging window. In general we found that motility data generated with the imaging windows tested quite faithfully represented the whole GC.

When we compared the three in vivo data sets to each other after normalizing data acquisition to a common time step (37–45 sec), window thickness (30 µm), and minimum track length (10 min), we observed that they displayed slightly different velocities (Figure S6). The reasons for this are unclear but likely represent technical variations between the three groups (see Discussion). These differences formed the basis for adjusting the relative programmed chemotaxis velocity for *GC B-cells* between the three groups.

### Data Analysis

All data analysis was performed using custom scripts with the student version of MatLab R2010a (MathWorks). Graphs were generated using either MatLab or GraphPad Prism 5 (GraphPad Software). The motility coefficient (M) was derived from the equation M = x^2^/6t, where x is the slope (x/t^1/2^) calculated from regression analysis of the linear portion (1.5–3 min^1/2^) of the mean displacement (x) versus the square root of time (t^1/2^) analysis [10], [26], [27].

Visual representation of PathSim2 output was generated using ParaView 3.6.2 (Kitware). Animations were produced with Photoshop and Flash CS5 (Adobe).

## Supporting Information

Figure S1 **Chemotaxis driven** ***lymphocyte*** **movement.** **(A)** An illustration depicting the model of chemotaxis. At each time step, *chemokine* molecules bind a *lymphocytes'* surface (black dots), resulting in a single maximum concentration point (yellow star). A *lymphocyte* will always attempt to travel in the direction of the highest concentration (arrow), and will pursue the target direction for a programmed amount of time, termed the persistence time (∼1–2 min). This represents the average time it takes for an immune cell to re-orient itself in response to a new chemokine gradient. During movement, bound *chemokines* are internalized while new molecules continue to accumulate on the surface. **(B)** A cartoon depicting *lymphocyte* movement in tissue. *Lymphocytes* are incompressible (i.e., in response to external forces, *cells* can change shape but their total volume remains constant) but are able to change shape (aspect ratio). Movement in a crowded environment is only possible if there is sufficient room. **(C)** Tonsil histology slice depicting the dense cellular environment of a GC (stained for B-cell marker CD20). Original magnification: 40X.
(TIF)

Figure S2 **Random walk analysis of experimental (Schwickert et al.) and simulation output.** **(A–B)** 10 min trajectory of a tracks from (n = 100) from **(A)** Schwickert et al. and **(B)** PathSim2. **(C)** In vivo data from Schwickert et al. (n = 310; M = 10.11 µm^2^min^−1^). **(D)** PathSim2 data (n = 3247; M = 10.66 µm^2^min^−1^). The green line is the best fit regression line to the data points (red bars, SD). Note the initial super linear behavior reflecting directed movement. The blue dashed line is the predicted best-fit for true random walk. At later times observed behavior approximates true random walk (linear over time^1/2^). **(E)** Linear regression analysis (in the form of y = b*x) yields a regression coefficient (b) of 1.0142 (95% confidence intervals: 1.0037, 1.0246)
(TIF)

Figure S3 **Random walk analysis of experimental (Hauser et al.) and simulation output.** **(A–B)** 10 min trajectory of a tracks from (n = 100) from (A) Hauser et al. and (B) PathSim2. **(C)** In vivo data from Hauser et al. (n = 483; M = 5.90 µm^2^min^−1^). **(D)** PathSim2 data (n = 1407; M = 5.65 µm^2^min^−1^). The green line is the best fit regression line to the data points (red bars, SD). Note the initial super linear behavior reflecting directed movement. The blue dashed line is the predicted best-fit for true random walk. At later times observed behavior approximates true random walk (linear over time^1/2^). (E) Linear regression analysis (in the form of y = b*x) yields a regression coefficient (b) of 0.9615 (95% confidence intervals: 0.9575, 0.9654)
(TIF)

Figure S4 **Experimental measurements are biased against detecting longer track lengths.** The actual observed cumulative cell track lengths are shown for all three in vivo data sets as a percentage over time (solid lines). For comparison, the Schwickert et al. (n = 188) and Hauser et al. (n = 217) data sets were examined using the criteria of Allen et al. (n = 400). That is, over a 30 min analysis, each cell track must remain in the imaging window for a minimum of 10 min to be included. Observed track lengths predicted by the simulation are shown for each of the three experimental conditions: Allen et al., n = 3741; Schwickert et al., n = 1903; Hauser et al., n = 716 (dashed lines). Note that the distribution of track lengths for each in vivo study is predicted by the simulation to vary with the size of the imaging window used. In each case, the observed distribution is further skewed towards shorter track lengths due to technical limitations in track reconstruction.
(TIF)

Figure S5 **The sensitivity of** ***GC B-cell*** **inter-zonal crossing to changes in parameters.** *Lymphocyte* tracks are not constrained by an imaging window and span the entire GC. (NB: inter-zonal crossing rates should not be compared directly to Figure 11, as that data was constrained by imaging windows.) Each analysis was for 30 min and was performed in triplicate from independent GC simulations (error bars indicate SEM). **(A)** The percentage of *centroblasts* that remain in the LZ for cell division, rather than crossing into the DZ, is varied and the effect this has on the crossing frequency is determined (n = ∼5500). 0% *centroblast* division in the LZ represents the traditional cyclic re-entry GC model, and crossing rates are compatible with previously published estimates [18]. (Full comparison to previous estimates requires a 60 min imaging session and a crossing frequency derived directly from agent state changes.) **(B)** The crossing frequency of *GC B-cells* (n = ∼3000–5000) over varying cell cycle lengths. For each cell cycle length analyzed, equal time is spent as a *centroblast* and *centrocyte.*
(TIF)

Figure S6 **Comparison of average instantaneous velocity from in vivo data.** All three data sets were normalized to common experimental parameters (time step of 37–45 sec, minimum track length of 10 min, and an imaging window thickness of 30 µm) and re-analyzed over 10-min. Allen et al. (n = 89), Schwickert et al. (n = 75), Hauser et al. (n = 227).
(TIF)

Figure S7 **Influence of** ***lymphocyte*** **packing density on zonal** ***chemokine*** **gradients.** *Chemokine* gradients are shown for **(A,C)** the default *lymphocyte* packing density (i.e., a densely-packed environment) and **(B,D)** an environment sparsely-populated with *lymphocytes*. **(A,B)** shows the gradient for the LZ (*CXCL13*) and **(C,D)** for the DZ (*CXCL12*). While the magnitude of the *CXCL13* (B) and *CXCL12* (D) concentrations have increased under sparse packing conditions, the overall gradients are relatively unaffected; the *CXCL13* gradient points in towards the follicle, while the *CCL21* gradient points out towards the extrafollicular zone. This is a result of *chemokine* diffusion throughout the tissue from the sites of production.
(TIF)

Video S1 **Basic Tonsil Unit.** A two-dimensional slice through an empty BTU is shown, rotating in three-dimensions. For all PathSim2 graphical visualizations, we display a cross-sectional slice through the tissue, orientated with the epithelium facing up (*epithelial cells* are shown in white). The *FRC* network is shown in orange, while the *FDC* network (denoting the *B-cell* follicle) is shown in yellow.
(MP4)

Video S2 **Primary follicle homeostasis.** The video begins with an empty BTU. As time progresses, *lymphocytes* enter the tissue. Colors: *naive B-cells* (red) and *naive CD4/8*. Driven by chemotaxis, *naive B-cells* migrate to the *FDC* populated follicle in response to *CXCL13*, while *memory B-cells* and *T-cells* remain in the *FRC* populated extrafollicular region in response to *CCL21/19*. *Cells* remain in the BTU for ∼12–24 hours, at which point they change their chemotaxis preference to the exit *chemokine* S1P and actively leave via efferent lymphatics at the bottom of the tissue (exiting *naive B-cells* shown in yellow).
(MP4)

Video S3 **Development of a mature GC.** The video begins with a naive follicle at homeostasis. It then follows the expansion of a GC initiated by 3 *GC founder B-cells* (yellow) to the production of a mature GC consisting of anatomically accurate MZ, LZ and DZ. *Naive B-cells* (red) highlight the MZ surrounding the GC. The zonal structure of the GC begins to emerge after day 3 of the GCR, and remains stable over the course of the animation, as *centroblasts* (DZ, pink) and *centrocytes* (LZ, orange) cycle between zones. *Follicular T-helpers* are visible in the LZ (light blue).
(MP4)

Table S1 **Model parameters.** This table lists and discusses relevant agent parameters used in our model. Experimental references are cited where applicable.
(DOC)
