# Supplementary figures and images for: Chemotaxis in Densely Populated Tissue Determines Germinal Center Anatomy and Cell Motility: A New Paradigm for the Development of Complex Tissues
Source: PLoS One. 2011 Dec 1;6(12):e27650. doi: 10.1371/journal.pone.0027650 (PMC3228727; doi:10.1371/journal.pone.0027650)

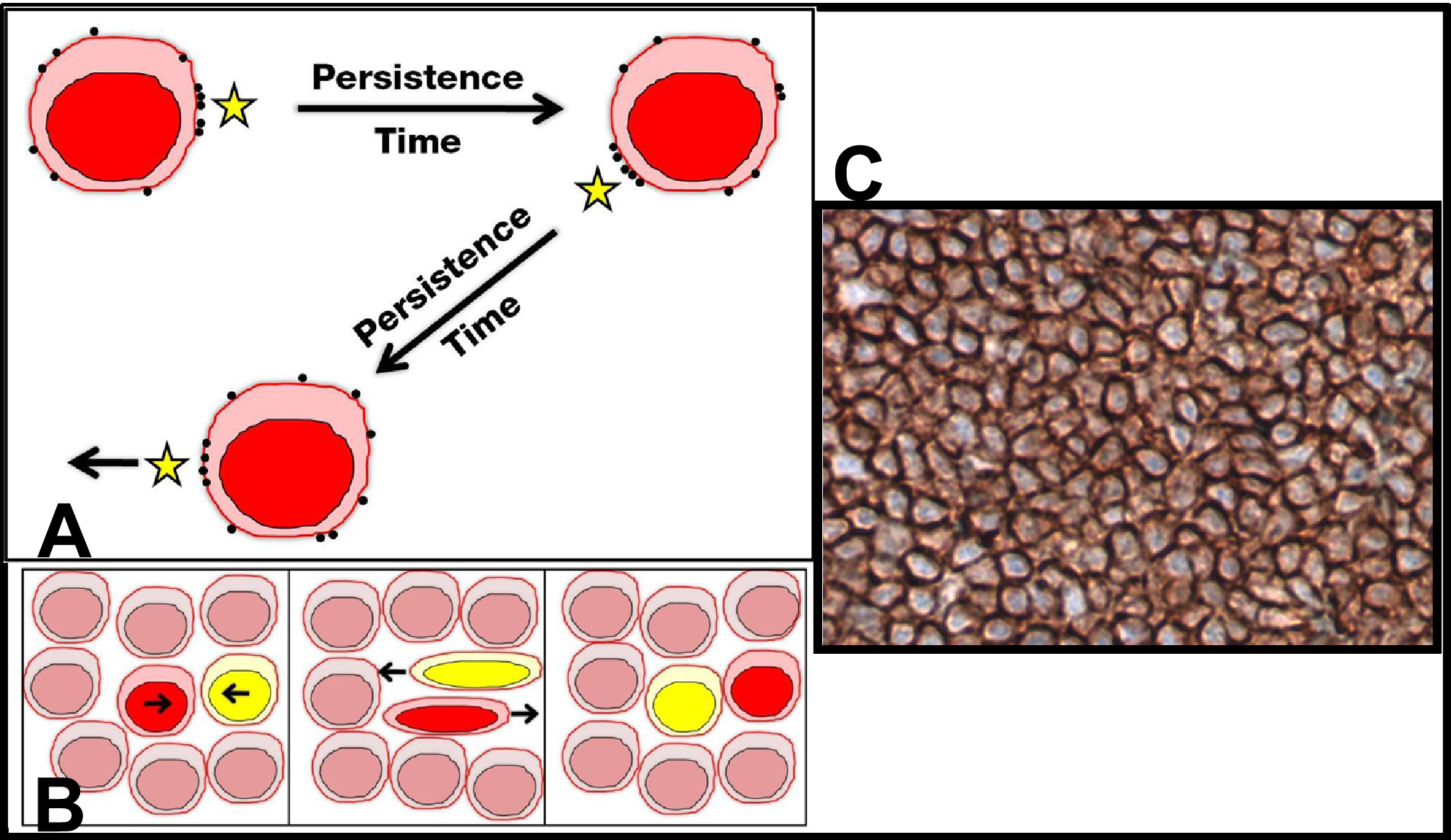

Supplement: Figure S1 — Chemotaxis driven lymphocyte movement. (A) An illustration depicting the model of chemotaxis. At each time step, chemokine molecules bind a lymphocytes' surface (black dots), resulting in a single maximum concentration point (yellow star). A lymphocyte will always attempt to travel in the direction of the highest concentration (arrow), and will pursue the target direction for a programmed amount of time, termed the persistence time (∼1–2 min). This represents the average time it takes for an immune cell to re-orient itself in response to a new chemokine gradient. During movement, bound chemokines are internalized while new molecules continue to accumulate on the surface. (B) A cartoon depicting lymphocyte movement in tissue. Lymphocytes are incompressible (i.e., in response to external forces, cells can change shape but their total volume remains constant) but are able to change shape (aspect ratio). Movement in a crowded environment is only possible if there is sufficient room. (C) Tonsil histology slice depicting the dense cellular environment of a GC (stained for B-cell marker CD20). Original magnification: 40X. (TIF) [file pone.0027650.s001.tif]

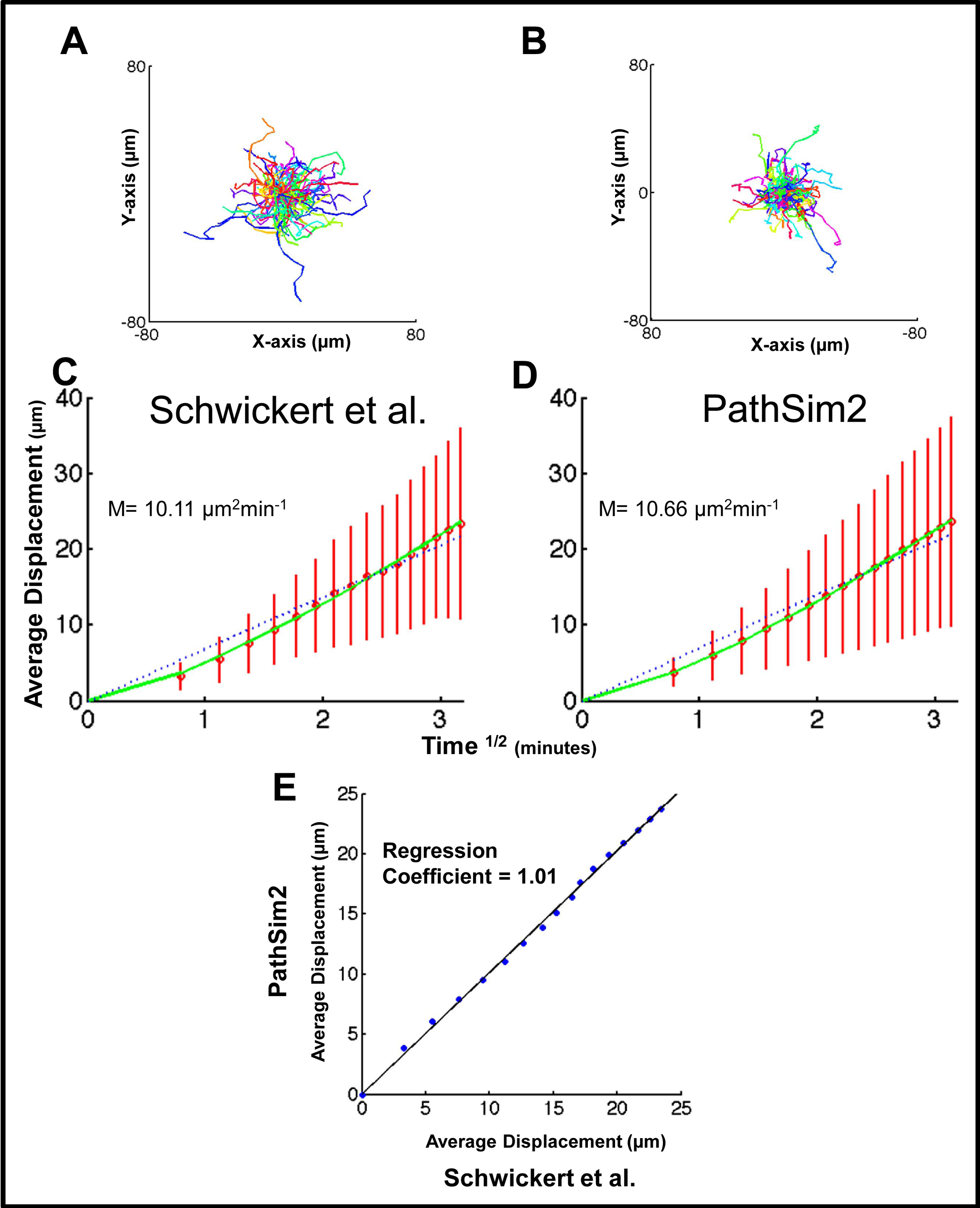

Supplement: Figure S2 — Random walk analysis of experimental (Schwickert et al.) and simulation output. (A–B) 10 min trajectory of a tracks from (n = 100) from (A) Schwickert et al. and (B) PathSim2. (C) In vivo data from Schwickert et al. (n = 310; M = 10.11 µm2min−1). (D) PathSim2 data (n = 3247; M = 10.66 µm2min−1). The green line is the best fit regression line to the data points (red bars, SD). Note the initial super linear behavior reflecting directed movement. The blue dashed line is the predicted best-fit for true random walk. At later times observed behavior approximates true random walk (linear over time1/2). (E) Linear regression analysis (in the form of y = b*x) yields a regression coefficient (b) of 1.0142 (95% confidence intervals: 1.0037, 1.0246) (TIF) [file pone.0027650.s002.tif]

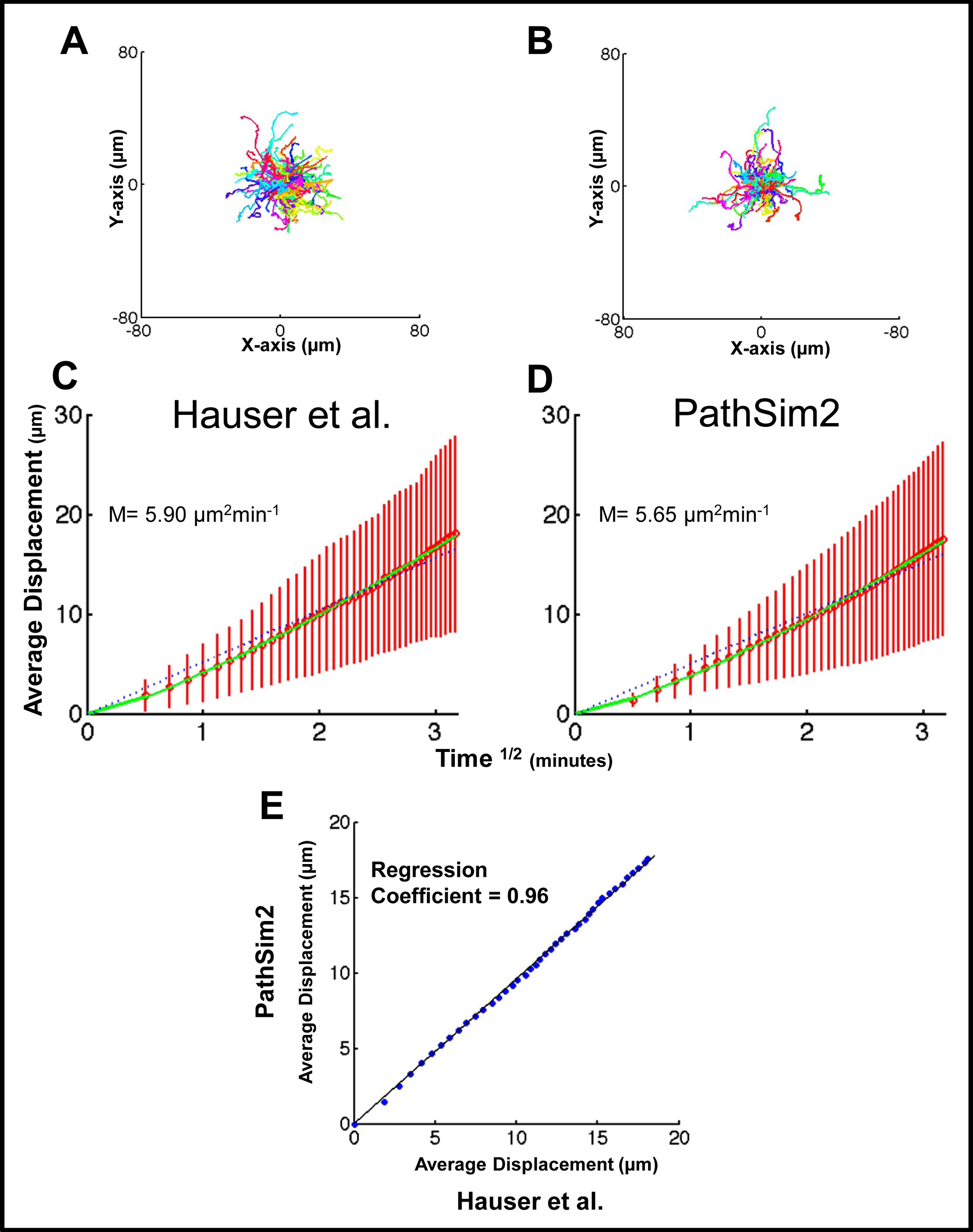

Supplement: Figure S3 — Random walk analysis of experimental (Hauser et al.) and simulation output. (A–B) 10 min trajectory of a tracks from (n = 100) from (A) Hauser et al. and (B) PathSim2. (C) In vivo data from Hauser et al. (n = 483; M = 5.90 µm2min−1). (D) PathSim2 data (n = 1407; M = 5.65 µm2min−1). The green line is the best fit regression line to the data points (red bars, SD). Note the initial super linear behavior reflecting directed movement. The blue dashed line is the predicted best-fit for true random walk. At later times observed behavior approximates true random walk (linear over time1/2). (E) Linear regression analysis (in the form of y = b*x) yields a regression coefficient (b) of 0.9615 (95% confidence intervals: 0.9575, 0.9654) (TIF) [file pone.0027650.s003.tif]

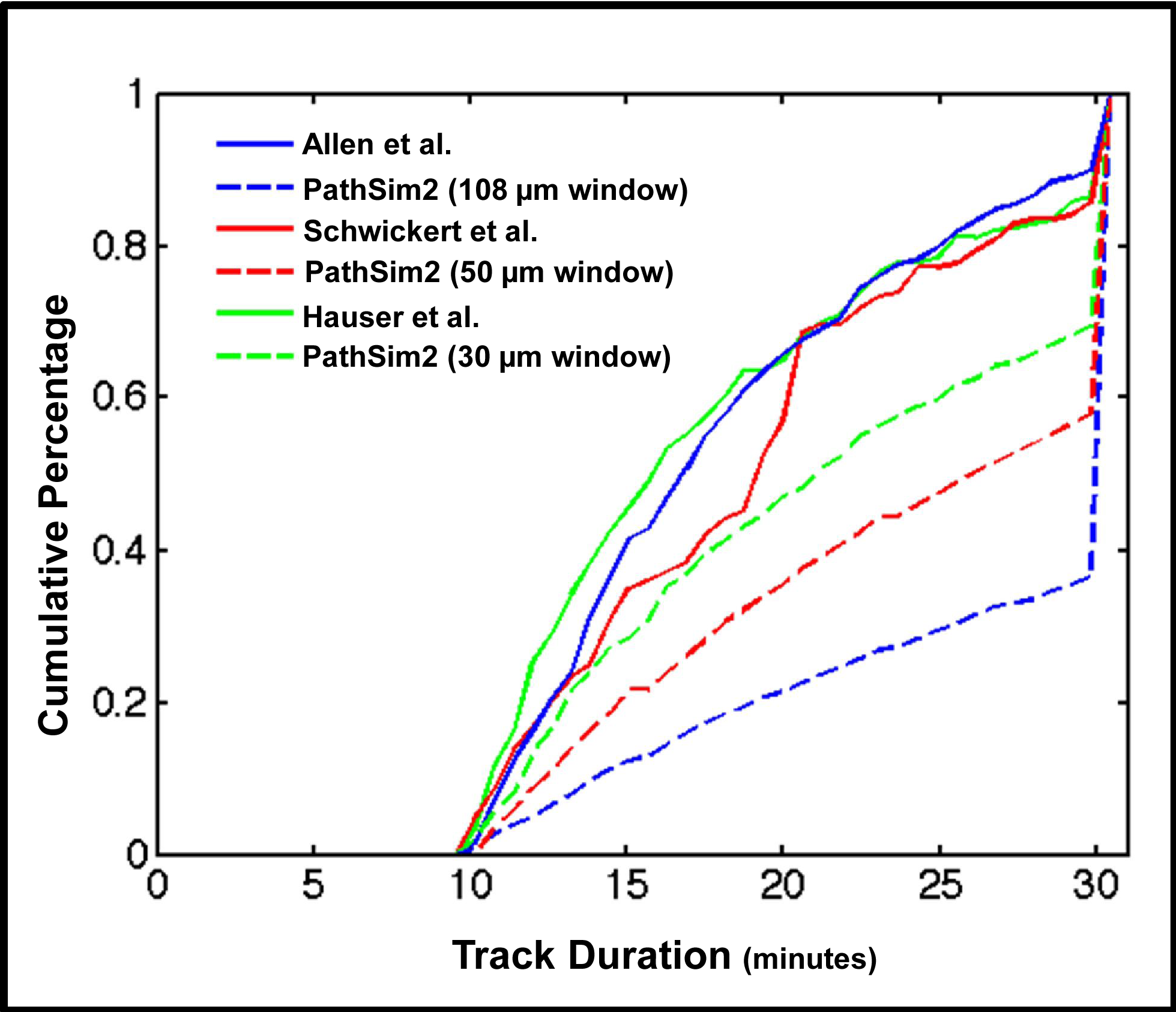

Supplement: Figure S4 — Experimental measurements are biased against detecting longer track lengths. The actual observed cumulative cell track lengths are shown for all three in vivo data sets as a percentage over time (solid lines). For comparison, the Schwickert et al. (n = 188) and Hauser et al. (n = 217) data sets were examined using the criteria of Allen et al. (n = 400). That is, over a 30 min analysis, each cell track must remain in the imaging window for a minimum of 10 min to be included. Observed track lengths predicted by the simulation are shown for each of the three experimental conditions: Allen et al., n = 3741; Schwickert et al., n = 1903; Hauser et al., n = 716 (dashed lines). Note that the distribution of track lengths for each in vivo study is predicted by the simulation to vary with the size of the imaging window used. In each case, the observed distribution is further skewed towards shorter track lengths due to technical limitations in track reconstruction. (TIF) [file pone.0027650.s004.tif]

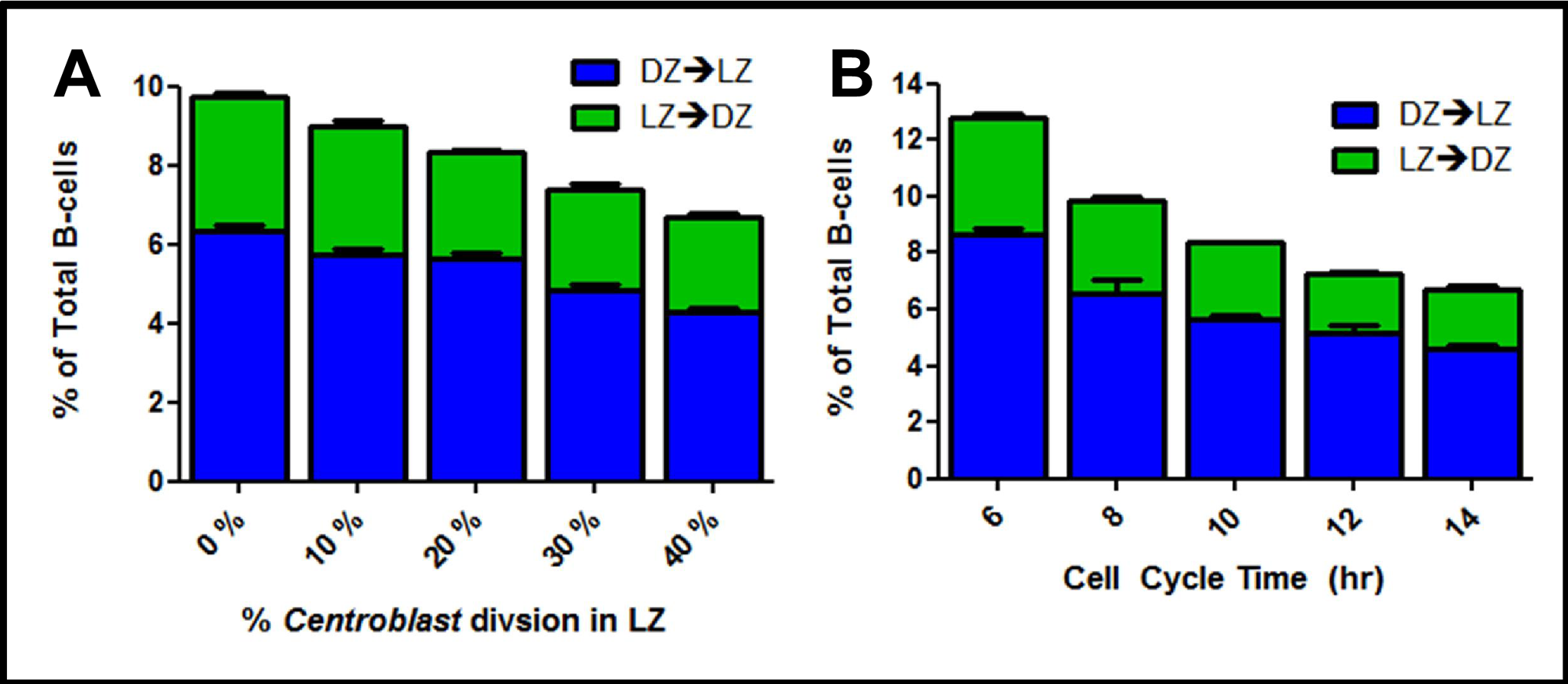

Supplement: Figure S5 — The sensitivity of GC B-cell inter-zonal crossing to changes in parameters. Lymphocyte tracks are not constrained by an imaging window and span the entire GC. (NB: inter-zonal crossing rates should not be compared directly to Figure 11, as that data was constrained by imaging windows.) Each analysis was for 30 min and was performed in triplicate from independent GC simulations (error bars indicate SEM). (A) The percentage of centroblasts that remain in the LZ for cell division, rather than crossing into the DZ, is varied and the effect this has on the crossing frequency is determined (n = ∼5500). 0% centroblast division in the LZ represents the traditional cyclic re-entry GC model, and crossing rates are compatible with previously published estimates [18]. (Full comparison to previous estimates requires a 60 min imaging session and a crossing frequency derived directly from agent state changes.) (B) The crossing frequency of GC B-cells (n = ∼3000–5000) over varying cell cycle lengths. For each cell cycle length analyzed, equal time is spent as a centroblast and centrocyte. (TIF) [file pone.0027650.s005.tif]

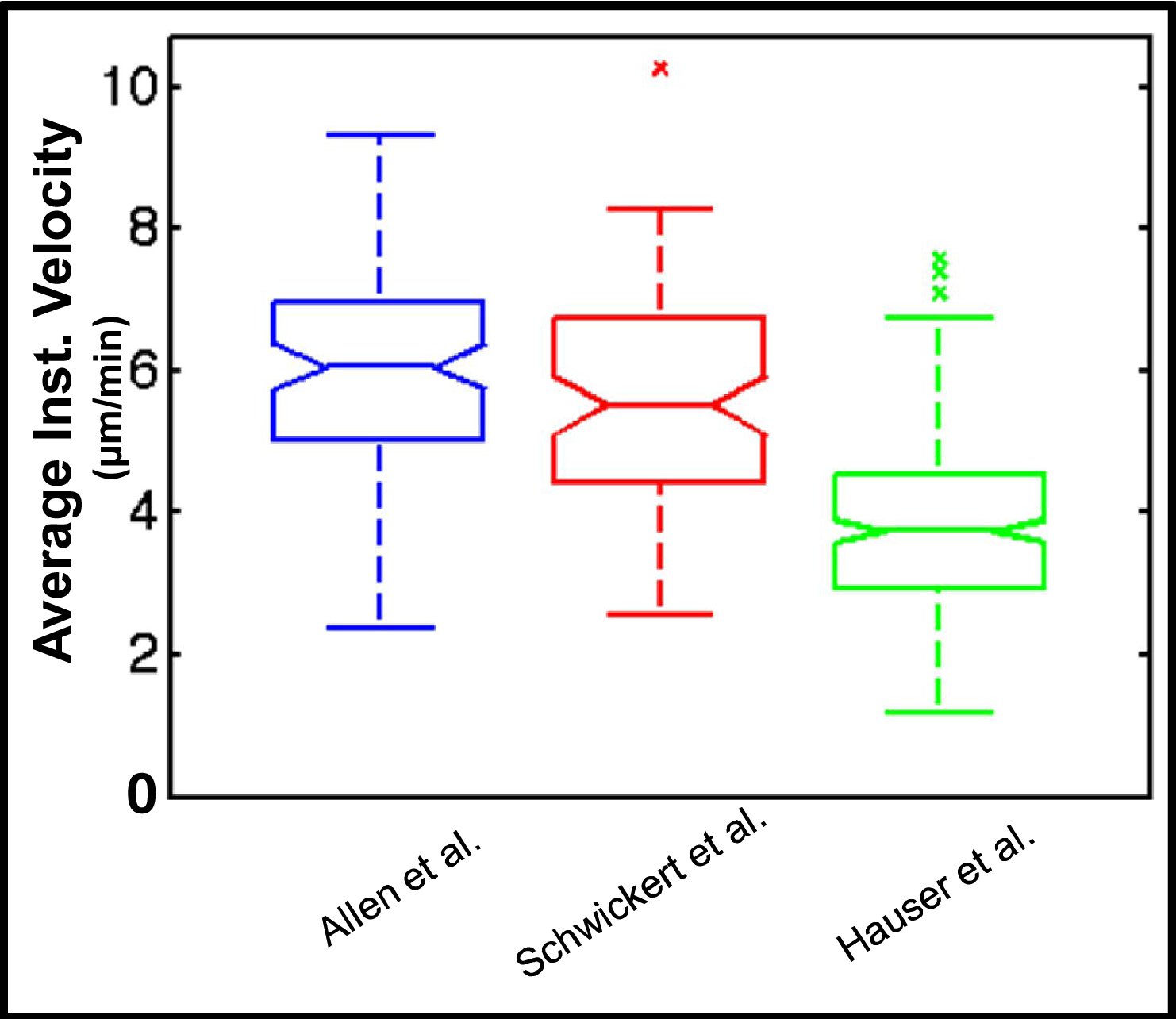

Supplement: Figure S6 — Comparison of average instantaneous velocity from in vivo data. All three data sets were normalized to common experimental parameters (time step of 37–45 sec, minimum track length of 10 min, and an imaging window thickness of 30 µm) and re-analyzed over 10-min. Allen et al. (n = 89), Schwickert et al. (n = 75), Hauser et al. (n = 227). (TIF) [file pone.0027650.s006.tif]

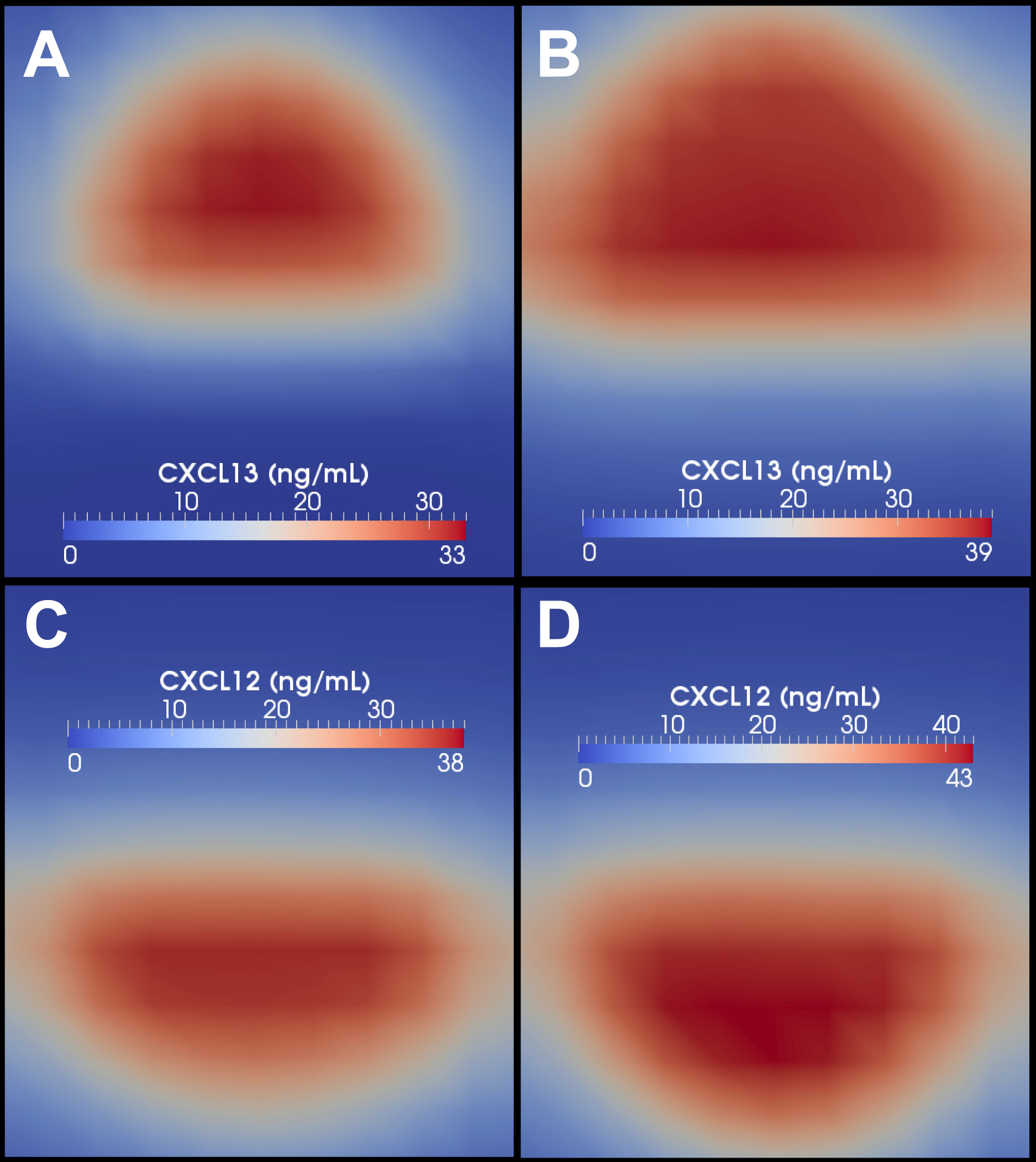

Supplement: Figure S7 — Influence of lymphocyte packing density on zonal chemokine gradients. Chemokine gradients are shown for (A,C) the default lymphocyte packing density (i.e., a densely-packed environment) and (B,D) an environment sparsely-populated with lymphocytes. (A,B) shows the gradient for the LZ (CXCL13) and (C,D) for the DZ (CXCL12). While the magnitude of the CXCL13 (B) and CXCL12 (D) concentrations have increased under sparse packing conditions, the overall gradients are relatively unaffected; the CXCL13 gradient points in towards the follicle, while the CCL21 gradient points out towards the extrafollicular zone. This is a result of chemokine diffusion throughout the tissue from the sites of production. (TIF) [file pone.0027650.s007.tif]
