# Supplementary material for: Chemotaxis in Densely Populated Tissue Determines Germinal Center Anatomy and Cell Motility: A New Paradigm for the Development of Complex Tissues
Source: PLoS One. 2011 Dec 1;6(12):e27650. doi: 10.1371/journal.pone.0027650 (PMC3228727; doi:10.1371/journal.pone.0027650)
Supplement: Table S1 — Model parameters. This table lists and discusses relevant agent parameters used in our model. Experimental references are cited where applicable. (DOC) [file pone.0027650.s011.doc]

**Supplemental Table:**

**Table S1: Model** Parameters

| **Agent** | **Parameter** | **Value** | **Class^a^** | **Notes** |
| --- | --- | --- | --- | --- |
| ***B-cell*** |  |  |  |  |
| *Naive* | diameter | 7 µm | exp. |  |
|  | chemotaxis - naive follicle (*CXCL13)* | 17 µm min^-1^ | est. | This parameter describes chemotaxis driven speed, but encapsulates both innate velocity and an agent’s competitive fitness (see text). Agent chemotaxis parameters were set, such that the resulting observed velocity was comparable to in vivo measurements. |
|  | chemotaxis - GC (*CXCL13)* | 8.5 µm min^-1^ | sim. | A low chemotaxis parameter within a GC reflects the inability of *naive B-cells* to efficiently compete for space within an activated follicle. See Figure 4 for the effect that varying this parameter relative to GC B-cells has on GC architecture. |
|  | time in LN (responsive to *CXCL13)* | 18 hr (±6 hr) | exp. | *Lymphocyte* retention time in the BTU is within the range of published estimates (12-24 hr). |
| *All GC B-cells* | survival outside of *FDC* network | ~1 hr | est. | This reflects the observation that GC B-cells quickly undergo apoptosis in vitro, and that this can be rescued by culturing with FDCs. In our model at homeostasis, death of GC B-cells is entirely due to failure to receive positive selection. Thus, while this parameter serves as an upper limit to GC growth, it does not contribute to GC homeostasis. |
| *GC Founder B-Cell* | diameter | 10 µm | exp. |  |
|  | # of *GC Founder B-Cells* | 3 | exp. | This parameter controls the initial number of *GC founder B-cells* that seed the GC and initiate the GCR. |
|  | division time | 6 hr (±1 hr) | exp. | This parameter determines the division time of *GC founder B-cells* during the initial expansion phase of the GCR. |
|  | Length of expansion phase | 69-75 hr | exp. | This parameter controls the duration of *GC founder B-cell* proliferation. *Centrocytes* exit the expansion phase during a 6 hr window to minimize the synchronization of *GC B-cell* populations. |
| *GC B-cell* | average cell cycle time | ~10 hr | exp. | This parameter represents the average time it takes a *GC B-cell* to complete the cell cycle. As different values have been reported in the literature, we have determined the effect that varying this parameter has on output (see Figure S5B). |
|  | chemotaxis parameter – speed (Allen et al.) | 17 µm min^-1^ (simulation default) | exp. | This parameter was chosen to best fit the observed velocity in the Allen et al. experimental data. |
|  | chemotaxis parameter – speed (Schwickert et al.) | 13 µm min^-1^ | exp. | This parameter was chosen to best fit the observed velocity in the Schwickert et al. experimental data. |
|  | chemotaxis parameter – speed (Hauser et al.) | 8.5 µm min^-1^ | exp. | This parameter was chosen to best fit the observed velocity in the Hauser et al. experimental data. |
| *Centroblast* | diameter | 10 µm | exp. |  |
|  | chemotaxis parameter – specificity | *CXCL12* | exp. | *Centroblasts,* while still able to respond to *CXCL13*, preferentially respond to *CXCL12*. |
|  | division time | 5 hr (±1 hr) | exp. | This parameter was chosen as an average of available in vivo measurements. See Figure S5B for the effect that varying this parameter has on output. |
| *Centrocyte* | diameter | 8 µm | exp. |  |
|  | chemotaxis – specificity | *CXCL13* | exp. | *Centrocytes* specifically respond to *CXCL13.* |
|  | length of positive selection in the LZ | ~ 5 hr (±1 hr) | exp. | This parameter represents the average time it takes a *centrocyte* to receive positive selection and initiate division, completing the cell cycle. See Figure S5B for the effect that varying this parameter has on output. |
|  | amount of FDC-antigen required for positive selection | 1.0 unit of FDC-antigen | sim. | *Centrocytes* must see 1.0 unit of FDC-antigen to become positively selected. This parameter was arbitrarily set to 1.0. The rate and extent of positive selection is controlled by the rate of FDC-antigen production by *LZ FDCs* (see below). |
|  | probability of recycling to *centroblast* (p_CB_) | p_CB_ = 0.8 | exp. | The parameter determines the probability of a selected *centrocyte* recycling back into a *centroblast*, rather than leaving the GC as non-specified output. This parameter has been previously determined by mathematical modeling. |
|  | probability of remaining in the LZ for *centroblast* division (p_LZ_) | p_LZ_ = 0.2 | est. | Based on in vivo measurements, we estimated ~20% of centrocytes remain in the LZ while undergoing cell division. As this was a rough approximation, we have determined the effect that varying this parameter has (see Figure S5A). |
| ***T-cell*** |  |  |  |  |
| all *naive T-cells* | diameter | 7 µm | exp. |  |
|  | time in LN (responsive to *CCL21)* | 18 hr (±6 hr) | exp. |  |
|  | chemotaxis parameter (*CCL21)* | 17 µm min^-1^ | est. | Aside from *follicular T-helpers,* all T-cells in the model are responsive to *CCL21*. |
| *follicular T-helper* | diameter | 8 µm | exp. |  |
|  | chemotaxis parameter (*CXCL13*) | 17 µm min^-1^ (simulation default) | sim. | This parameter was set to match the speed of *GC B-cells.* This is required to allow both *follicular T-helpers* and *GC B-cells* to compete for the same limited space in the follicle. This parameter was decreased along with the speed of *GC B-cells* when matching experimental data. |
|  | # of *follicular T-helpers* in mature GC | ~ 500 (~5% total GC) | exp. | In vivo estimates of # of follicular T-helpers range from 5-20% of total GC. |
| ***Stromal Network*** |  |  |  |  |
| All *stromal cells* | # of stromal cells in each element | 8 | sim. | This is a simulation parameter used to distribute stromal cells throughout each element. They should not be thought of as individual *FDCs,* but rather a continuous network. |
|  | total space occupied in each element by *stromal cells* | 25 % | est. | This parameter is an estimation of space occupied by the stromal network within an intact lymph node and is used to generate physiological cell density. All *stromal cells* are assumed to occupy the same amount of space. |
|  | production of stromal *chemokines* (*CXCL13/12/21*) | 1e5 cytokine molecules min^-1^ | sim. | *Stromal cell* *chemokine* production is regulated by a negative feedback loop. Production is empirically set to generate a locally fluctuating cytokine gradient (see text). |
|  | max. free cytokine threshold | 0.9e5 cytokine molecules | sim. | This parameter sets the maximum threshold that turns off cytokine production by *stromal cells* within an element. |
|  | min. free cytokine threshold | 0.65e5 cytokine molecules | sim. | This parameter sets the minimum threshold that turns on cytokine production by *stromal cells* within an element. |
|  | target *chemokine* concentration (free *chemokine* in solution) | ~35 ng/mL | sim. | At this concentration, effectively 10^4^ *chemokine* receptors are bound on each *lymphocyte* within the densely packed BTU. This concentration will be significantly lower than that measured in vivo, as it refers only to free cytokine in solution. |
| *LZ FDCs* | total *LZ FDCs* | 1260 | sim. | Activation of the FDC network induces differentiation of *LZ FDCs* and *DZ stromal cells.* The LZ size is influenced by the space needed for the DZ (see below). |
|  | rate of FDC-presented antigen (positive selection) | 0.0125 units of antigen min^-1^ | sim. | This parameter controls the rate and extent of positive selection within the LZ. This encapsulates both successful interaction with antigen and sufficient T-cell help. *Centrocytes* not selected die via apoptosis. The value was empirically determined to produce stable GC populations at equilibrium. Higher values will result in increased *GC B-cell* death, as *cells* than cannot fit in the follicle die via apoptosis. Our default value ensures that all *GC B-cell* death results from a failure to receive positive selection, and no *cells* are dying from lack of space in the follicle. |
| *DZ stromal cells* | total *DZ stromal cells* | 884 | sim. | Activation of the FDC network induces differentiation of *LZ FDCs* and *DZ stromal cells.* The size of the DZ is set based on the number of *centroblasts* at equilibrium. |
| ***Chemokine*** |  |  |  |  |
|  | *chemokine* diffusion coefficient | 1000 µm ^2^ min^-1^ | exp. | This parameter is derived by: 1) assuming a chemokine size of ~10 kDa, 2) approximating the effective diameter to be ~0.1 µm, 3) using the viscosity of water, 4) using a standard formula for the translational diffusion coefficient . This parameter is within the range of previously published values . |
|  | surface *chemokine* internalized / min | 1/3 | sim. | This parameter controls *chemokine* turnover on an agent’s surface, which influences the agent’s target direction. This is a rough approximation representing receptor-mediated chemokine internalization, and not based on a specific published estimate. |
|  | *Lymphocyte p*ersistence time | 1-2 min | exp. | This parameter reflects the average length of time required for an immune cell to re-orient itself in response to a new chemokine gradient (in vitro experiment). |
|  | Max. aspect ratio of *lymphocyte* during movement | 8.0 | sim. | This parameter controls a *lymphocyte’s* shape changes during movement, where aspect ratio of a *lymphocyte* is defined as longest dimension / smallest dimension. This is an approximation of cell flexibility during movement. An agent’s ability to change aspect ratio (as depicted in Figure S1) is the essential component of the novel flow computations that allows agents to efficiently move through the tissue and around other agents. However, in our model, the actual magnitude of the aspect ratio is less important, as similar movement results are seen with lower aspect ratios. |
|  | *Chemokine* “consumption” rate | 0.175 / *lymphocyte* volume min^-1^ | sim. | Within each element, *chemokine* is “eaten” (internalized) by *lymphocytes* that are specific for it. The exact number of *chemokine* molecules “eaten” is further influenced by the *lymphocyte* density within each element. This parameter was empirically determined to produce *chemokine* gradients that are compatible with biological data/observations. |
|  | *Chemokine* non-specific degradation | 0.2 * *Chemokine* “consumption” rate | sim. | Within each element, *chemokine* is non-specifically degraded by all agents (including inert agents) not specific for it. This encapsulation represents non-specific degradation within tissue. This parameter was empirically determined to produce *chemokine* gradients that are compatible with biological data/observations. |
| **Misc.** |  |  |  |  |
| BTU | element dimensions | 31.75 µm (l) x 31.75 µm (w) x 31.75 µm (h) | sim. |  |
|  | mesh dimensions | 317.5 µm (l) x 317.5 µm (w) x 603.25 µm (h) | sim. | As the mesh is comprised of 31.75 µm^3^, this corresponds to 10 elements (l) x 10 elements (w) x 19 elements (h). |
|  | follicle dimensions | An ellipsoid with an equatorial radius of ~150 µm | sim. | This follicle dimension was chosen to hold a mature GC, including the MZ. The MZ sits at the edge of the follicle, pushed up against the extra-follicular region. |
| GC | Total *GC cells* | ~10^4^ | exp. | This value includes both *GC B-cells* as well as *follicular T-helper cells*. This estimate is for an average mouse GC, to which we are directly comparing our data. By our own labs estimates, this size GC is small but within the range of human tonsillar GCs. |
|  | GC Diameter | ~240 µm | exp. | This is the observed diameter of a mature GC in PathSim2. It is a result of the total GC-cells, and not predetermined. This measurement is consistent with published values. |

^a^ Note on Class: Experimental parameters are derived from personal/published measurements and are believed to be accurate. Estimated parameters, while also taken from the literature, are approximated with less certainty. Simulation parameters are empirically determined to produce desired output.

References:

1. Allen CD, Okada T, Tang HL, Cyster JG (2007) Imaging of germinal center selection events during affinity maturation. Science 315: 528-531.

2. Schwab SR, Cyster JG (2007) Finding a way out: lymphocyte egress from lymphoid organs. Nat Immunol 8: 1295-1301.

3. Liu YJ, Joshua DE, Williams GT, Smith CA, Gordon J, et al. (1989) Mechanism of antigen-driven selection in germinal centres. Nature 342: 929-931.

4. Lindhout E, Mevissen ML, Kwekkeboom J, Tager JM, de Groot C (1993) Direct evidence that human follicular dendritic cells (FDC) rescue germinal centre B cells from death by apoptosis. Clin Exp Immunol 91: 330-336.

5. Koopman G, Keehnen RM, Lindhout E, Newman W, Shimizu Y, et al. (1994) Adhesion through the LFA-1 (CD11a/CD18)-ICAM-1 (CD54) and the VLA-4 (CD49d)-VCAM-1 (CD106) pathways prevents apoptosis of germinal center B cells. J Immunol 152: 3760-3767.

6. Jacob J, Kassir R, Kelsoe G (1991) In situ studies of the primary immune response to (4-hydroxy-3-nitrophenyl)acetyl. I. The architecture and dynamics of responding cell populations. J Exp Med 173: 1165-1175.

7. Liu YJ, Zhang J, Lane PJ, Chan EY, MacLennan IC (1991) Sites of specific B cell activation in primary and secondary responses to T cell-dependent and T cell-independent antigens. Eur J Immunol 21: 2951-2962.

8. Kroese FG, Wubbena AS, Seijen HG, Nieuwenhuis P (1987) Germinal centers develop oligoclonally. Eur J Immunol 17: 1069-1072.

9. Hauser AE, Junt T, Mempel TR, Sneddon MW, Kleinstein SH, et al. (2007) Definition of germinal-center B cell migration in vivo reveals predominant intrazonal circulation patterns. Immunity 26: 655-667.

10. Schwickert TA, Lindquist RL, Shakhar G, Livshits G, Skokos D, et al. (2007) In vivo imaging of germinal centres reveals a dynamic open structure. Nature 446: 83-87.

11. Allen CD, Ansel KM, Low C, Lesley R, Tamamura H, et al. (2004) Germinal center dark and light zone organization is mediated by CXCR4 and CXCR5. Nat Immunol 5: 943-952.

12. Victora GD, Schwickert TA, Fooksman DR, Kamphorst AO, Meyer-Hermann M, et al. (2010) Germinal center dynamics revealed by multiphoton microscopy with a photoactivatable fluorescent reporter. Cell 143: 592-605.

13. Meyer-Hermann M, Deutsch A, Or-Guil M (2001) Recycling probability and dynamical properties of germinal center reactions. J Theor Biol 210: 265-285.

14. Miller MJ, Wei SH, Parker I, Cahalan MD (2002) Two-photon imaging of lymphocyte motility and antigen response in intact lymph node. Science 296: 1869-1873.

15. Allen CD, Okada T, Cyster JG (2007) Germinal-center organization and cellular dynamics. Immunity 27: 190-202.

16. Arnold CN, Campbell DJ, Lipp M, Butcher EC (2007) The germinal center response is impaired in the absence of T cell-expressed CXCR5. Eur J Immunol 37: 100-109.

17. Haynes NM, Allen CD, Lesley R, Ansel KM, Killeen N, et al. (2007) Role of CXCR5 and CCR7 in follicular Th cell positioning and appearance of a programmed cell death gene-1high germinal center-associated subpopulation. J Immunol 179: 5099-5108.

18. Kelsoe G (1996) The germinal center: a crucible for lymphocyte selection. Semin Immunol 8: 179-184.

19. Bajenoff M, Egen JG, Koo LY, Laugier JP, Brau F, et al. (2006) Stromal cell networks regulate lymphocyte entry, migration, and territoriality in lymph nodes. Immunity 25: 989-1001.

20. Berg HC (1983) Random walks in biology. Princeton, N.J.: Princeton University Press. ix, 142 p. p.

21. Young ME, Carroad PA, Bell RL (1980) Estimation of Diffusion Coefficients of Proteins. Biotechnology and Bioengineering 22: 947-955.

22. Zigmond SH, Levitsky HI, Kreel BJ (1981) Cell polarity: an examination of its behavioral expression and its consequences for polymorphonuclear leukocyte chemotaxis. J Cell Biol 89: 585-592.

23. Gerisch G, Keller HU (1981) Chemotactic reorientation of granulocytes stimulated with micropipettes containing fMet-Leu-Phe. J Cell Sci 52: 1-10.

24. MacLennan IC (1994) Germinal centers. Annu Rev Immunol 12: 117-139.
